# Characterizing preoperative domain-specific performance on the Montreal Cognitive Assessment and exploring its associations with adverse outcomes

**DOI:** 10.1007/s44254-026-00181-2

**Published:** 2026-06-17

**Authors:** Ellene Yan, Yasmin Alhamdah, Aparna Saripella, Eric Cheuk, Sazzadul Islam, David He, Keera N. Fishman, Leif Erik Lovblom, Maria Carmela Tartaglia, David F. Tang-Wai, Jean Wong, Frances Chung

**Affiliations:** 1 Department of Anesthesia and Pain Management, Toronto Western Hospital, University Health Network, University of Toronto, Toronto, ON M5T 2S8 Canada; 2 Institute of Medical Science, Temerty Faculty of Medicine, University of Toronto, Toronto, ON Canada; 3 Temerty Faculty of Medicine, University of Toronto, Toronto, ON Canada; 4 Krembil Brain Institute, University Health Network, Toronto, ON Canada; 5 Neuropsychology & Cognitive Health, Baycrest Hospital, Toronto, ON Canada; 6 Ontario Shores Centre for Mental Health Sciences, Whitby, ON Canada; 7 Department of Anesthesia and Pain Management, Mount Sinai Hospital, University of Toronto, Toronto, ON Canada; 8 Biostatistics Department, University Health Network, Toronto, ON Canada; 9 Division of Neurology, Department of Medicine, University of Toronto, Toronto, ON Canada; 10 Women’s College Hospital, Toronto, ON Canada

**Keywords:** Cognitive impairment, Older patients, Adverse outcomes, Surgery, Montreal Cognitive Assessment, Anesthesia

## Abstract

**Purpose:**

The Montreal Cognitive Assessment (MoCA) is a validated screening tool for cognitive impairment (CI) in surgical populations that assesses multiple cognitive domains. The primary objective of this report was to characterize preoperative domain-specific performance on the MoCA in older surgical patients. The secondary objectives were to explore preoperative characteristics and adverse outcomes associated with poorer domain-specific performance.

**Methods:**

This was a post hoc analysis of the Detection of Cognitive Impairment (Detect CI) study. The MoCA was administered preoperatively to assess seven cognitive domains: executive/visuospatial function, naming, attention, language, abstraction, delayed recall, and orientation. It was scored out of 30, with higher education-adjusted MoCA scores indicating better cognitive performance and scores $\le$ 25 classifying probable CI. Adverse outcomes were collected in 382 participants at 30 days and 379 participants at 90 days postoperatively.

**Results:**

The 382 participants (median age, 73 years [IQR, 68, 77]; 58% female) had a mean MoCA score of 25.9 $\pm$ 2.8, with 35% screening positive for CI. Participants with probable CI performed significantly poorer across all seven cognitive domains (executive/visuospatial function, naming, attention, language, abstraction, delayed recall, and orientation) than those without CI. Having $\le$ 12 years of education was associated with poorer executive/visuospatial function, naming, language, abstraction, and delayed recall. Of all cognitive domains, exploratory analyses showed that only orientation was associated with 30-day adverse outcomes after adjusting for age, sex, education level, and/or American Society of Anesthesiologists status and within-domain multiplicity. Each one-point decrease in orientation score was associated with a longer length of stay (β = 0.5, 95% confidence interval [95% CI] 0.2–0.8; *P *$=$ 0.012) and higher adjusted odds of postoperative delirium (adjusted odds ratio [aOR] = 11.4, 95% CI 2.6–46.8; *P *$=$ 0.004), complications (aOR = 3.1, 95% CI 1.3–8.9; *P *$=$ 0.035), non-home discharge (aOR = 4.1, 95% CI 1.5–11.4; *P *$=$ 0.014), and composite adverse outcomes (aOR = 5.6, 95% CI 1.9–23.9; *P *$=$ 0.014). Orientation remained associated with postoperative delirium after adjusting for multiplicity both within and across MoCA domains (aOR = 11.4, 95% CI 2.6–46.8; *P *$=$ 0.031).

**Conclusions:**

This analysis characterized preoperative domain-specific performance on the MoCA in older adults, with participants screening positive for CI exhibiting poorer performance across all cognitive domains. Exploratory findings suggested that orientation difficulties may be associated with early adverse outcomes, with postoperative delirium showing the most robust association.

**Supplementary Information:**

The online version contains supplementary material available at 10.1007/s44254-026-00181-2.

## Introduction

Approximately one third of individuals may be affected by neurological disorders such as dementia or major neurocognitive disorders during their lifetime, which collectively rank as the leading cause of disability worldwide [1, 2]. As the population ages and grows, the global number of individuals with dementia is projected to rise by 166% between 2019 and 2050, highlighting an urgency to improve and tailor care for this vulnerable population [3].

Individuals with cognitive impairment (CI), ranging from mild cognitive impairment (MCI) to dementia, may be less resilient to stressors associated with surgery and anesthesia [4]. With lower cognitive reserve, they have a higher risk of adverse postoperative outcomes such as delirium, discharge to assisted care, and mortality [5–7]. Despite their heightened risk, preoperative assessment of brain health was documented in less than 1% of patients, primarily due to time constraints, workflow issues, and a lack of available tools in electronic health records [8]. As a result, CI remains undiagnosed in 37% of elective surgical patients, complicating recovery and raising safety concerns [9].

The Montreal Cognitive Assessment (MoCA), a cognitive screening tool validated in surgical populations, allows for early detection of patients at risk of CI and adverse postoperative outcomes [10, 11]. Importantly, the MoCA’s ability to assess multiple cognitive domains provides a more nuanced understanding of cognitive vulnerabilities beyond a binary classification of CI [12]. Domain-specific performance helps to better characterize the etiology of CI, stage severity, inform possible functional limitations, and identify areas that would benefit from targeted care strategies [12]. Specifically, impairments related to memory and executive function have been identified as risk factors for postoperative delirium [13–15]. Although the MoCA cannot replace comprehensive neuropsychological batteries, it offers an efficient first step to recognize early signs of CI perioperatively [12].

Despite previous literature supporting the construct validity of the MoCA domain classifications when compared with neuropsychological batteries, limited studies have explored its utility in characterizing the cognitive profile of older surgical populations and predicting adverse events [16]. Preoperative characterization of cognitive vulnerabilities can promote risk stratification, facilitate education with patients and their families about brain health, and guide proactive perioperative planning. The primary objective of this exploratory post hoc analysis was to characterize the distributions of preoperative MoCA domain scores in older adults undergoing elective non-cardiac surgery. The secondary objectives were to explore (1) the degree of correlation among the domain scores, (2) preoperative characteristics associated with domain-specific performance, and (3) associations between domain-specific performance and adverse outcomes at 30 and 90 days postoperatively.

## Materials and methods

This study was a post hoc analysis of the multicenter, longitudinal, prospective cohort Detection of Cognitive Impairment (Detect CI) Study (NCT06030765; registered at ClinicalTrials.gov) [17]. Research Ethics Boards (REB) approval was obtained from the University Health Network and Mount Sinai Hospital (approval numbers 22-5810 and 22-0194-E), and all recruited participants provided informed consent. The manuscript is presented in accordance with the STROBE reporting checklist (Supplementary Table S1).

Participants were recruited consecutively from August 30, 2023, to July 30, 2024, at the preoperative clinics of Toronto Western Hospital and Mount Sinai Hospital, Toronto, Ontario, Canada. All participants were $\ge$ 65 years old, scheduled for elective non-cardiac surgery with an expected length of stay (LOS) of $\ge$ 1 day, and had $\ge$ 8 years of formal education. Although the standard 1-point educational adjustment in MoCA scoring was applied to participants with $\le$ 12 years of schooling, this adjustment likely remains insufficient to account for the influence of educational attainment on test performance in older adults [18, 19]. Thus, we only included participants with $\ge$ 8 years of education to further minimize the risk of misclassification. We excluded individuals with diagnosed major neurocognitive disorders, uncontrolled psychiatric disorders, hearing/visual impairment, inability to write or hold a pen, or those scheduled for vascular surgery or neurosurgery. Detailed study methodology has been published previously [17].

### Cognitive performance on the Montreal Cognitive Assessment

One to 30 days before surgery, research personnel with MoCA training and certification administered the MoCA (version 8.1) at the preoperative clinics [10]. The MoCA is a 10-min pencil and paper cognitive screening tool designed for detecting MCI [10, 11]. It has been validated and widely adopted across various settings, with the cut-off $\le$ 25 showing 87% sensitivity and 72% specificity for identifying CI in surgical populations [10, 11].

The MoCA is a screening tool that assesses multiple cognitive domains: (1) executive/visuospatial function (maximum score of 5), (2) naming (maximum score of 3), (3) attention (maximum score of 6), (4) language (maximum score of 3), (5) abstraction (maximum score of 2), (6) delayed recall (maximum score of 5), and (7) orientation (maximum score of 6) [10]. Specifically, the executive/visuospatial function component consists of three individual tasks: making a trail by alternating between numbers and letters in sequence, copying a cube, and drawing a clock. Attention is examined using forward and backward digit span, vigilance, and serial 7 subtraction. The language domain assesses sentence repetition and verbal fluency. Delayed recall evaluates the number of words participants can recall without cues. Lastly, orientation items include the date, month, year, day, place (name of the hospital), and city. Total MoCA scores range from 0 to 30, with higher scores indicating better cognitive performance. To mitigate the influence of education on cognitive performance, those with $\le$ 12 years of education received one additional point as per routine practice [10]. Given the heterogeneous classifications of MoCA cognitive domains across the literature, the domain classification and scoring approach used in this analysis was based on the original MoCA framework to promote consistency [10, 20].

In this study, CI classification was based on participants’ MoCA scores, rather than comprehensive neuropsychological batteries and clinical diagnoses. Specifically, those scoring $\le$ 25 on the MoCA were classified as screening positive for CI (“CI group”) whereas those scoring $>$ 25 were considered to have normal cognition (“No-CI group”).

### Variables for secondary objectives

To examine preoperative characteristics associated with MoCA performance, clinically relevant variables such as age, sex, education level, American Society of Anesthesiologists (ASA) physical status, history of transient ischemic attack (TIA) or stroke, obstructive sleep apnea (OSA), frailty, clinically significant functional disability, anxiety/depression, and sleep quality were collected from electronic medical records (EMR) or self-reports from patients. The following 30- and 90-day postoperative outcomes were collected: delirium, all-cause complications, LOS, non-home discharge, emergency room (ER) visits, 30-day readmission, and mortality. Specifically, all-cause complications included cardiovascular, respiratory, gastrointestinal, renal, neurological, and surgical complications. If participants experienced any of the adverse outcomes listed previously, they were classified as having composite adverse outcomes.

These adverse outcomes were identified through reviewing EMR by research personnel blinded to participants’ MoCA performance. To ensure all adverse events were captured, research personnel also asked participants directly (or their informants if the participants were unavailable) about the presence of any adverse events via the telephone or an online REDCap survey (http://www.project-redcap.org) [21]. To identify postoperative delirium during participants’ hospital stays, the Confusion Assessment Method was administered by nursing staff every shift and supplemented by the Chart-based Delirium Identification Instrument by research personnel [22, 23].

### Statistical analysis

Descriptive statistics for demographics, preoperative characteristics including MoCA scores and their domain scores, and clinical outcomes are summarized as mean ± standard deviation, median [interquartile range], or count (percentage). To compare the demographics and preoperative characteristics between participants with and without a positive MoCA screen ($\le$ 25 *vs *$>$ 25 MoCA scores), we used Fisher's exact, chi-square, Student’s t-test, or Mann–Whitney U test, depending on the variable’s distribution.

For the first secondary objective, the degree of correlation among MoCA scores across the seven cognitive domains was examined, with Spearman’s rho (ρ) of |0.1–0.3| indicating weak correlations, |0.4–0.6| moderate correlations, and |0.7–0.9| strong correlations [24]. For the second and third secondary objectives, which aimed to explore preoperative characteristics and adverse outcomes associated with poorer domain-specific performance, both univariable and multivariable regression analyses were conducted as described below. All estimates are presented alongside their respective 95% confidence intervals (95% CIs). Multicollinearity was low as assessed by the variance inflation factor, and goodness-of-fit was confirmed using the Hosmer–Lemeshow test.

For the second secondary objective, we explored clinically relevant preoperative characteristics associated with lower scores on each cognitive domain. All multivariable linear regression models included the following preselected covariates due to their associations with cognitive functioning: age, sex, education level, ASA status, previous TIA/stroke, OSA, frailty, clinically significant functional disability, anxiety/depression, and sleep quality. To maintain consistency, the same covariates were included across the regression models examining the seven MoCA domains.

For the third secondary objective, the association between each unit decrease in domain-specific score (indicating poorer cognitive performance) and binary adverse outcomes was quantified using logistic regression analyses and presented as odds ratios (ORs). Linear regression analyses were performed for the continuous outcome of LOS. To account for its non-normal distribution and zero values, a value of one was added to LOS followed by a logarithmic transformation, and the estimates are expressed as beta-coefficients (β). All covariates included in the multivariable regression models were preselected based on clinical relevance, with the inclusion of the full model guided by the event count to avoid overfitting. As educational attainment is an important confounding variable, all multivariable analyses were at least minimally adjusted for education level to mitigate its influence on cognitive performance, except mortality due to its low event count (*n* = 2). For 30-day outcomes of all-cause complications, LOS, non-home discharge, ER visits, and composite adverse outcomes, multivariable analyses were adjusted for education level, age, sex, and ASA status. Multivariable analyses for 90-day outcomes of all-cause complications, ER visits, and composite adverse outcomes were adjusted for education level and age.

In this exploratory analysis, we applied Benjamini-Hochberg (BH) corrections instead of more conservative approaches to avoid missing potentially meaningful associations while balancing the risk of type I and type II errors. BH corrections were applied to the multivariable analyses to adjust for multiple comparisons under the assumption of independence or positive dependence [25, 26]. BH corrections were applied within each MoCA domain, treating each domain as a family of tests to control the false discovery rate at 0.05, and this was repeated for all seven domains. Given that multiplicity may further exist across MoCA domains, we conducted an additional sensitivity analysis by applying BH corrections both within and across domains as one single batch. This served as a more conservative approach, ensuring that the overall false discovery rate was not underestimated.

To further address residual confounding, the following sensitivity analyses were conducted. Given the subjective and broad nature of ASA classification, models that originally included ASA status were replaced with specific comorbidities closely associated with CI. Specifically, previous TIA/stroke, hypertension, OSA, and anxiety/depression were included instead of ASA status in separate sensitivity analyses, while retaining education level, age, and sex as covariates. Statistically significant multivariable models for orientation were further adjusted for preoperative CI status. Lastly, E-values were calculated to assess the robustness of the observed associations between orientation and adverse outcomes to potential unmeasured confounding [27].

The sample size was determined a priori based on the primary and secondary objectives of the original observational study, which examined the diagnostic accuracy of two ultra-rapid cognitive screening tools against the MoCA in older surgical patients and the perioperative trajectory of functional disability among those with and without probable CI [17]. No formal sample size calculation was performed specifically for this exploratory post hoc analysis. The present report included all participants who completed the MoCA preoperatively and subsequently underwent their scheduled procedures (*n* = 382). There were no missing data for key variables, namely MoCA scores and 30-day clinical outcomes. Specifically, clinical outcomes were collected for all 382 participants at 30 days and 379 participants at 90 days. All statistical analyses were performed in R, version 4.4.2, with two-tailed hypothesis tests and an α-level of 0.05.

## Results

### Study population

**Fig. 1:**
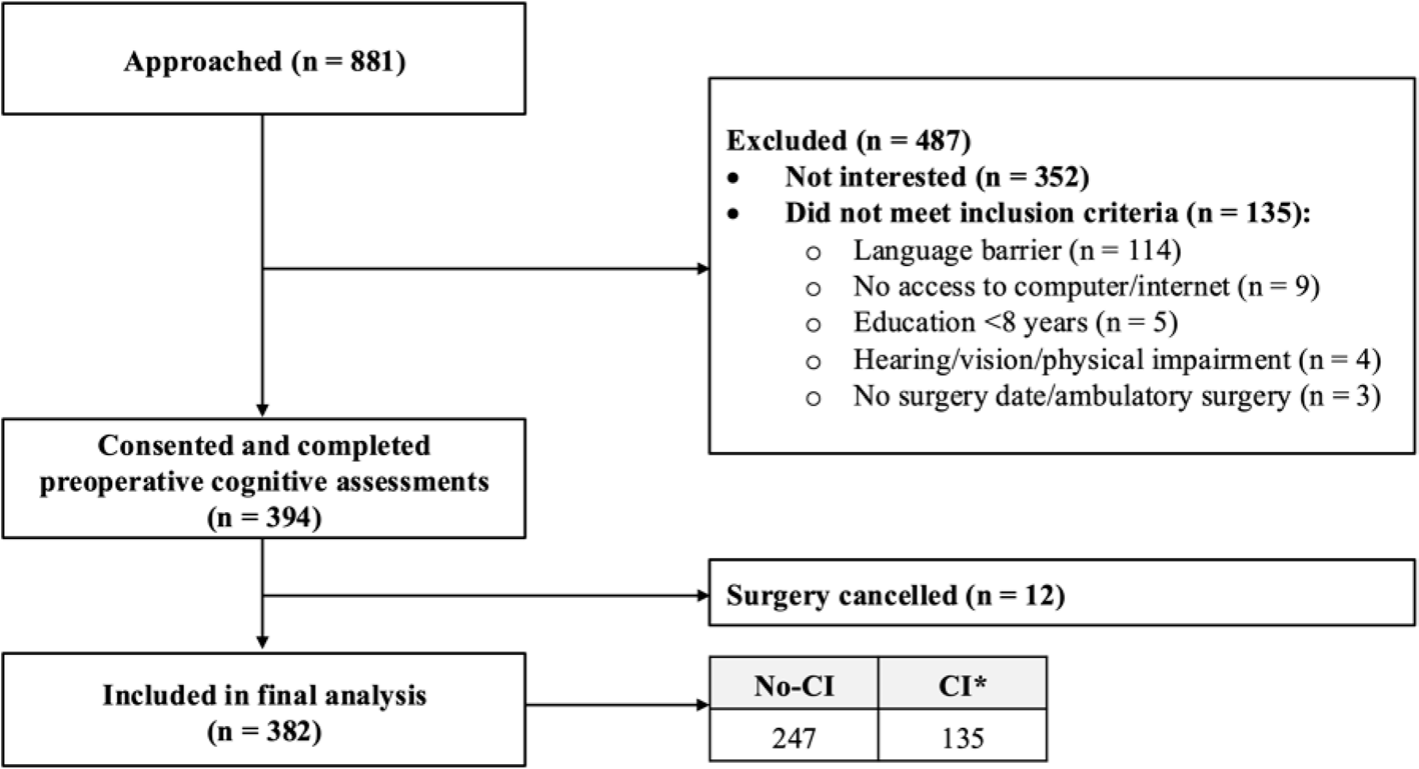
Study flowchart. *CI* cognitive impairment. *Classification of CI and No-CI was based on the established Montreal Cognitive Assessment (MoCA) cut-off of $\le$ 25. Reproduced and adapted from Yan et al. 2025, The utility of the Ascertain Dementia Eight-item Questionnaire (AD8) and Mini-Cog in detecting cognitive impairment in older surgical patients – The Detect CI study, published by Elsevier Inc [17] under the CC BY-NC license (https://creativecommons.org/licenses/by-nc/4.0/)

**Fig. 2:**
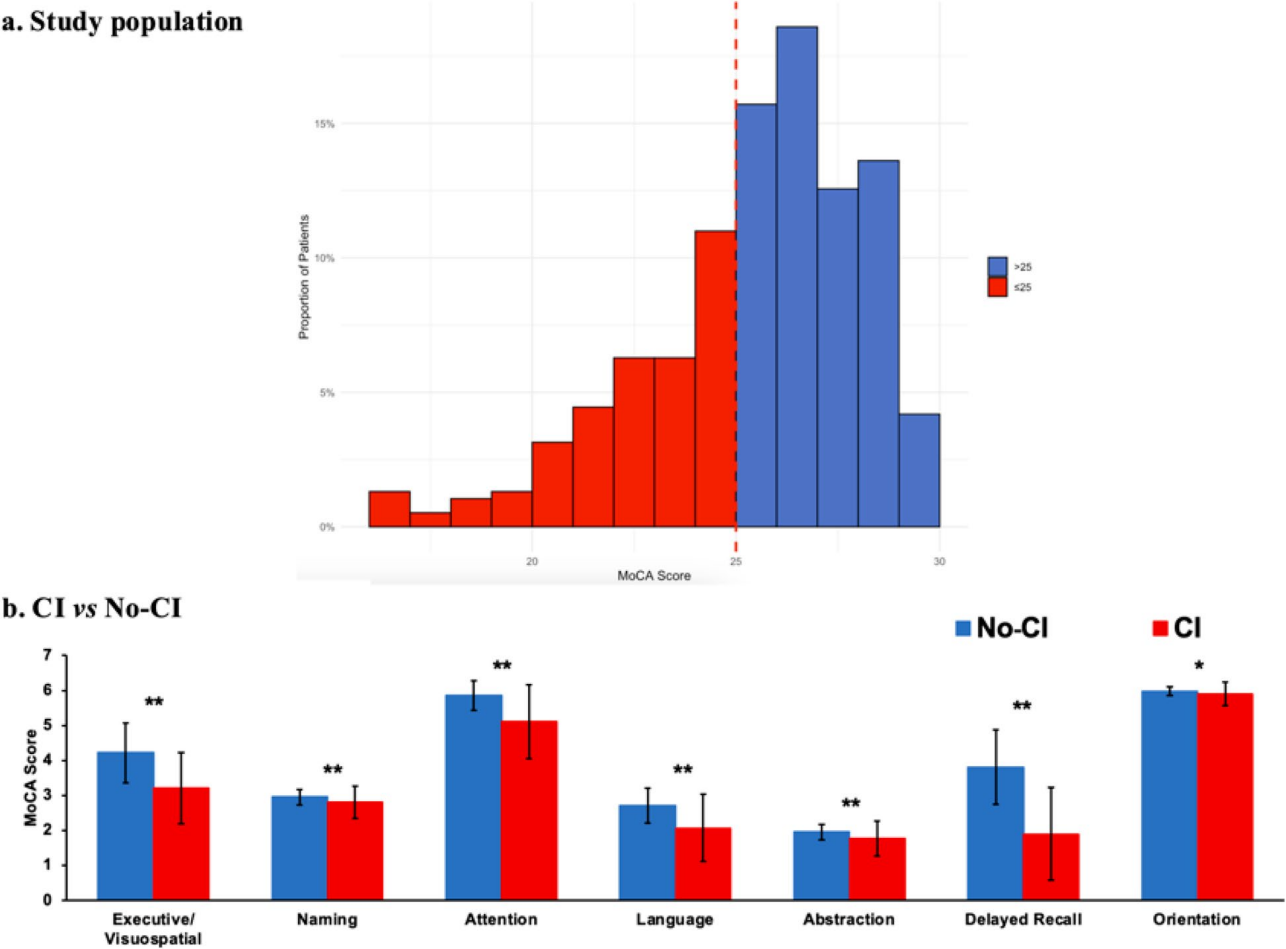
Distribution of MoCA scores in the study population (**a**) and in CI vs No-CI participants (**b**). *CI* cognitive impairment, *MoCA* Montreal Cognitive Assessment. *Classification of CI and No-CI was based on the established MoCA cut-off of $\le$ 25, indicated by the dashed red line. Values expressed as mean and error bars represented standard deviation. Scores range from 0–5 for executive/visuospatial function, 0–3 for naming, 0–6 for attention, 0–3 for language, 0–2 for abstraction, 0–5 for delayed recall, and 0–6 for orientation (lower scores indicate poorer cognitive performance). **P-values of ≤ 0.001, *P-values of ≤ 0.05 between CI and No-CI patients

The final analysis included 382 participants who completed the MoCA preoperatively and underwent surgery (Fig. 1). Of these participants, the mean MoCA score was 25.9 $\pm$ 2.8, with 35% screening positive for CI (i.e., $\le$ 25 on the MoCA) (Fig. 2a; Table 1). Participants with CI had a mean MoCA score of 22.9 $\pm$ 2.2. Compared with the No-CI group, the CI group was significantly older, reported fewer years of formal education, and had a higher proportion of individuals with a history of TIA or stroke (Table 1).

**Table 1:** Demographics and characteristics of the study population

Baseline characteristic	Total (*n* = 382)	No-CI (*n* = 247)	CI* (*n* = 135)	*P*-value
Age, years	73.0 [68.0–77.0]	72.0 [68.0–76.0]	73.0 [69.0–78.0]	0.022
Sex, female	221 (57.9)	146 (59.1)	75 (55.6)	0.57
Education, years	15.5 ± 2.9	15.9 ± 2.9	14.8 ± 2.8	$<$ 0.001
$\le$12 years	50 (13.1)	28 (11.3)	22 (16.3)	0.22
Ethnicity/Race^a^				
White	337 (88.2)	224 (90.7)	113 (83.7)	0.09
Asian	32 (8.4)	17 (6.9)	15 (11.1)	
Black	6 (1.6)	3 (1.2)	3 (2.2)	
Hispanic	4 (1.0)	2 (0.8)	2 (1.5)	
Other^b^	2 (0.5)	1 (0.4)	1 (0.7)	
Preferred not to answer	1 (0.3)	0 (0.0)	1 (0.7)	
BMI, kg/m^2^	29.1 ± 6.3	29.3 ± 6.3	28.7 ± 6.2	0.40
ASA Status				
I	0 (0.0)	0 (0.0)	0 (0.0)	0.37
II	49 (12.8)	35 (14.2)	14 (10.4)	
III	267 (69.9)	169 (68.4)	98 (72.6)	
IV	66 (17.3)	43 (17.4)	23 (17.0)	
Surgical Procedure				
Orthopedic	229 (59.9)	148 (59.9)	81 (60.0)	$>$ 0.99
General	48 (12.6)	33 (13.4)	15 (11.1)	
Urological	35 (9.2)	21 (8.5)	14 (10.4)	
Otolaryngological	32 (8.4)	20 (8.1)	12 (8.9)	
Gynecological	21 (5.5)	13 (5.3)	8 (5.9)	
Spinal	17 (4.5)	12 (4.9)	5 (3.7)	
Medical History				
OSA	80 (20.9)	57 (23.1)	23 (17.0)	0.21
Hypertension	218 (57.1)	137 (55.5)	81 (60.0)	0.45
CAD	38 (9.9)	22 (8.9)	16 (11.9)	0.46
Diabetes Mellitus	72 (18.8)	40 (16.2)	32 (23.7)	0.10
Asthma/COPD	62 (16.2)	42 (17.0)	20 (14.8)	0.68
TIA/Stroke	19 (5.0)	7 (2.8)	12 (8.9)	0.018
Anxiety/Depression	83 (21.7)	56 (22.7)	27 (20.0)	0.63
STOP-Bang	3.0 [2.0–4.0]	3.0 [2.0–4.0]	3.0 [2.0–5.0]	0.48
OSA ( $\ge$ 3)	263 (69.0)	167 (67.9)	96 (71.1)	0.59
MoCA score	25.9 ± 2.8 26 [25–28]	27.6 ± 1.2 27 [27–29]	22.9 ± 2.2 23 [22–25]	$<$ 0.001 $<$ 0.001

Values expressed as mean ± SD, median [interquartile range], or *n* (%) where appropriate

*Abbreviations*: *ASA* American Society of Anesthesiologists, *BMI* body mass index, *CAD* coronary artery disease, *CI* cognitive impairment, *COPD* chronic obstructive pulmonary disease, *MoCA* Montreal Cognitive Assessment, *OSA* obstructive sleep apnea, *SD* standard deviation, *STOP-Bang* Snoring, Tiredness, Observed apnea, High blood pressure, Body mass index, Age, Neck circumference, and Gender, *TIA* Transient ischemic attack

^a^Data for preferred not to answer were not included in *P*-value calculations

^b^Other ethnicity/race was comprised of mixed, Semitic, and Jewish

*Classification of CI and No-CI was based on the established MoCA cut-off of $\le$ 25. *P*-values of $\le$ 0.05 indicate statistical significance

Adapted from Yan et al. [17], The utility of the Ascertain Dementia Eight-item Questionnaire (AD8) and Mini-Cog in detecting cognitive impairment in older surgical patients – The Detect CI study, published by Elsevier Inc [17] under the CC BY-NC license (https://creativecommons.org/licenses/by-nc/4.0/)

### Primary descriptive analysis: distribution of MoCA scores across cognitive domains

Compared with the No-CI group, those with CI performed significantly poorer across all seven domains of the MoCA: executive/visuospatial function, naming, attention, language, abstraction, delayed recall, and orientation (Fig. 2b; Supplementary Table S2). Specifically, executive/visuospatial function, attention, and language domains were further stratified into individual items. The CI group had significantly lower mean scores across all individual items, except for vigilance (attention domain), than the No-CI group.

Intercorrelations among MoCA cognitive domain scores were examined across all participants (Supplementary Table S3). Although the correlations were weak overall, the strongest associations were observed between performance on attention and executive/visuospatial function, followed by attention and delayed recall, attention and abstraction, and language and executive/visuospatial function.

### Secondary exploratory analysis: preoperative characteristics associated with poorer domain-specific cognitive performance

Univariable and multivariable analyses explored preoperative factors associated with poorer performance in each cognitive domain assessed by the MoCA (Table 2; Supplementary Tables S4–10). An education level of $\le$ 12 years was associated with lower scores on five domains after accounting for age, sex, ASA status, previous TIA/stroke, OSA, frailty, clinically significant functional disability, anxiety/depression, and sleep quality. Specifically, lower education level was associated with poorer performance on executive/visuospatial function, naming, language, abstraction, and delayed recall but not attention or orientation.

**Table 2:** Multivariable analyses of preoperative factors statistically associated with MoCA scores

Cognitive domain and baseline characteristic	Comparison	Multivariable analysis^a^	
		Adjusted estimate (95% CI)	Adjusted* P*-value
*Executive/visuospatial function*			
Education	$\le$ 12 years *vs * $>$ 12	−0.34 (−0.65, −0.02)	0.037
*Naming*			
Education	$\le$ 12 years *vs * $>$ 12	−0.20 (−0.30, −0.10)	$<$0.001
*Attention*			
TIA/Stroke	Yes *vs* No	−0.53 (−0.91, −0.16)	0.005
*Language*			
Education	$\le$ 12 years *vs * $>$ 12	−0.43 (−0.67, −0.20)	$<$0.001
OSA	Yes *vs* No	0.27 (0.08, 0.47)	0.005
Clinically significant disability (WHODAS$\ge$35%)	Disability *vs* No Disability	−0.26 (−0.51, −0.01)	0.041
*Abstraction*			
Education	$\le$ 12 years *vs * $>$ 12	−0.18 (−0.28, −0.07)	0.001
Clinically significant disability (WHODAS$\ge$35%)	Disability *vs* No Disability	−0.115 (−0.227, −0.003)	0.044
*Delayed Recall*			
Age	+ 5 years	−0.17 (−0.31, −0.03)	0.015
Sex	Female *vs* Male	0.44 (0.14, 0.76)	0.005
Education	$\le$ 12 years *vs * $>$ 12	−0.54 (−0.99, −0.08)	0.022
*Orientation*			
Age	+ 5 years	−0.03 (−0.05, −0.01)	0.008

Estimates expressed as beta-coefficients for continuous outcomes. Total MoCA scores ranged from 0 to 30, with lower scores indicating poorer cognitive performance

*Abbreviation*: *OSA* obstructive sleep apnea, *TIA* transient ischemic attack, *WHODAS* World Health Organization Disability Assessment Schedule, *95% CI* 95% confidence interval

^a^Multivariable linear regression analyses adjusted for age, sex, education, ASA physical status, TIA/stroke, OSA, frailty, clinically significant functional disability, anxiety/depression, and sleep quality. Race/ethnicity, coronary artery disease, hypertension, and pain were not included to avoid statistical overfitting. *P*-values of $\le$ 0.05 indicated statistical significance

In addition to lower education, clinically significant functional disability was associated with poorer language and abstraction, while older age and male sex were associated with poorer delayed recall. Only advancing age was associated with lower orientation scores. While previous TIA/stroke were associated with poorer attention, OSA was associated with better language performance.

### Secondary exploratory analysis: associations between poorer domain-specific cognitive performance and adverse postoperative outcomes

For the secondary objective, associations between poorer domain-specific performance and adverse postoperative outcomes were explored. While 30-day clinical outcomes were collected for all participants (*n* = 382), data were unavailable for one participant at 90 days (Supplementary Table S11). Of all the cognitive domains assessed preoperatively (i.e., executive/visuospatial function, naming, attention, language, abstraction, delayed recall, and orientation), only orientation was significantly associated with adverse postoperative outcomes after adjusting for confounding variables of age, sex, education level, and/or ASA status and multiplicity within each MoCA domains (Table 3; Supplementary Tables S12–18). Notably, each unit decrease in orientation score was associated with higher adjusted odds of postoperative delirium by 11-fold, 30-day all-cause complications by threefold, non-home discharge by fourfold, and 30-day composite adverse outcomes by sixfold. Lower orientation score was also associated with a longer LOS (β = 0.50, 95% CI 0.17–0.82; *P *$=$ 0.012). As demonstrated by the sensitivity analysis, the association between orientation and postoperative delirium remained statistically significant after adjusting for multiplicity both within and across MoCA domains.

**Table 3:** Statistically significant multivariable associations between poorer MoCA performance and adverse outcomes

Clinical outcomes	Univariable analysis		Multivariable analysis^a^			
	Unadjusted estimate (95% CI)	Unadjusted* P*-value	Adjusted estimate (95% CI)	Adjusted *P*-value	Multiplicity-adjusted *P*-value (BH within domains)	Multiplicity-adjusted *P*-value (BH within/across domains)
*Executive/visuospatial function*						
Postoperative delirium	2.04 (1.00, 4.23)	0.047	2.41 (1.16, 5.18)	0.018	0.18	0.16
90-day ER visits	0.55 (0.27, 1.01)	0.08	0.48 (0.22, 0.91)	0.038	0.19	0.25
*Naming*						
30-day composite outcomes	0.48 (0.21, 0.97)	0.06	0.43 (0.18. 0.87)	0.029	0.17	0.21
*Attention*						
Non-home discharge	1.47 (1.06, 2.01)	0.018	1.49 (1.06, 2.06)	0.017	0.17	0.16
*Delayed Recall*						
Postoperative delirium	2.02 (1.19, 3.78)	0.014	2.25 (1.29, 4.42)	0.008	0.08	0.10
*Orientation*						
Postoperative delirium	8.94 (2.16, 34.49)	$<$ 0.001	11.38 (2.62, 46.77)	$<$ 0.001	0.004	0.031
30-day all-cause complications	3.24 (1.30, 9.30)	0.017	3.14 (1.25, 8.94)	0.020	0.035	0.16
LOS, days	0.51 (0.18, 0.84)	0.003	0.50 (0.17, 0.82)	0.003	0.012	0.09
Non-home discharge	4.03 (1.54, 10.66)	0.004	4.10 (1.46, 11.41)	0.006	0.014	0.10
30-day composite outcomes	6.26 (2.08, 27.27)	0.004	5.56 (1.89, 23.92)	0.006	0.014	0.10

Estimates expressed as odds ratios for binary outcomes or beta-coefficients for the continuous outcome of LOS (log-transformed), corresponding to each one unit decrease in MoCA subdomain score (lower scores indicates poorer performance). BH corrections were applied within each MoCA domain (BH within domains) and both within and across MoCA domains (BH within/across domains). All-cause complications included cardiovascular, respiratory, gastrointestinal, renal, neurological, and surgical complications. Composite adverse outcomes included postoperative delirium, all-cause complications, non-home discharge, ER visits, 30-day readmission and/or mortality

*Abbreviations*: *BH* Benjamini–Hochberg, *ER* emergency room, *LOS* length of stay, *95% CI* 95% confidence interval

^a^For outcomes of postoperative delirium and 30-day readmission, multivariable analyses were adjusted for education. Mortality was not adjusted due to low count for event. For 30-day outcomes of all-cause complications, length of stay, non-home discharge, emergency room visits, and composite adverse outcomes, multivariable analyses were adjusted for age, sex, education, and ASA physical status. For 90-day outcomes of all-cause complications, ER visits, and composite adverse outcomes, multivariable analyses were adjusted for age and education level. *P*-values of $\le$ 0.05 indicated statistical significance

The following associations did not remain significant after accounting for multiple comparisons within MoCA domains (Table 3). Namely, lower executive/visuospatial function and delayed recall scores were no longer associated with higher odds of postoperative delirium, and poorer attention did not remain associated with non-home discharge (Table 3). Additionally, the associations between poorer naming and executive/visuospatial function and lower adjusted odds of 30-day composite adverse outcomes and 90-day ER visits were no longer significant after multiplicity adjustment, respectively.

Additional sensitivity analyses were conducted to further address residual confounding. Among the statistically significant multivariable models that adjusted for ASA status as a general marker of health status, all associations remained significant after replacing ASA status with specific comorbidities (e.g., previous TIA/stroke, hypertension, OSA, and anxiety/depression) (Supplementary Tables S19). Furthermore, orientation remained significantly associated with postoperative delirium, 30-day all-cause complications, longer LOS, non-home discharge, and 30-day composite adverse outcomes after further adjustment for preoperative CI status in the multivariable models (Supplementary Tables S20). E-values suggested that an unmeasured confounder would need to be strongly associated with both poorer orientation and postoperative delirium to fully explain the observed association, suggesting robustness to unmeasured confounding. Compared with delirium, the associations between orientation and 30-day complications, non-home discharge, and composite adverse outcomes demonstrated more modest robustness (Supplementary Table S21).

## Discussion

In this exploratory post hoc analysis, over one in three older participants screened positive for CI (MoCA $\le$ 25) before surgery. The CI group performed significantly poorer across all domains of executive/visuospatial function, naming, attention, language, abstraction, delayed recall, and orientation than those without CI. Our secondary analyses showed that, of all domains, only orientation was associated with adverse outcomes after adjusting for age, sex, education level, and/or ASA status and multiplicity within each domain. The association between orientation and postoperative delirium remained significant after more conservative sensitivity analysis addressing multiplicity both within and across MoCA domains.

As the number of older surgical patients continues to increase, preoperative clinics should assess cognition to help identify high-risk patients with cognitive vulnerabilities, with pragmatic measures representing more feasible options [4]. These exploratory and hypothesis-generating findings highlight that older surgical patients screening positive for CI on the MoCA may frequently exhibit difficulties across multiple cognitive domains. Furthermore, orientation may warrant further investigation as a pragmatic approach to identify patients who may benefit from closer perioperative monitoring.

Patients with CI may exhibit deficits that vary by both the affected cognitive domain and the severity of impairment. Although cut-off scores on cognitive screening tools enable efficient classification of probable CI, domain-specific performance yields insights into the nature and extent of cognitive decline and possibly functional difficulties. Importantly, assessing multiple cognitive domains can capture subtle or early signs of cognitive decline, which may be missed by relying solely on dichotomous screening criteria for CI [28].

Patterns of domain-specific impairment on the MoCA may help clinicians identify subtypes and stages of CI [28]. For instance, Alzheimer’s disease has been associated with greater impairments in the MoCA domains of memory, attention, visuospatial function, and orientation, while those with vascular CI tend to exhibit attention-related difficulties [12]. Patterns can also reflect the progression of cognitive deficits as the disease advances. Moderate and severe domain-specific impairment may inform functional difficulties [28]. We found that participants with a positive MoCA screen exhibited global difficulties across executive/visuospatial function, naming, attention, language, abstraction, delayed recall, and orientation, rather than isolated deficits within specific cognitive domains. Impairment in memory and orientation may interfere with medication adherence, whereas executive function may impact one’s ability to manage finances, shop, and complete housekeeping tasks independently [28, 29]. Critically, early recognition of domain-specific impairment can help tailor cognitive and functional support, patient education, and post-discharge planning to proactively address one’s needs during surgical recovery.

In our secondary analysis, we found that poorer orientation scores on the MoCA were associated with adverse outcomes after surgery, including longer LOS β = 0.50, postoperative delirium (11-fold), complications (threefold), non-home discharge (fourfold), and composite adverse outcomes at 30 days (sixfold). More conservative adjustments, which considered multiplicity both within and across MoCA domains, revealed that lower orientation scores remained significantly associated with greater postoperative delirium but not other outcomes. It should be noted that most of our participants performed well on orientation, suggesting a ceiling effect that reduces sensitivity to detect subtle impairments and limits generalizability. Nevertheless, lower orientation scores were associated with greater odds of postoperative delirium. As impaired orientation often suggests moderate to severe CI, these heightened risks likely reflect perioperative vulnerability associated with more advanced cognitive dysfunction, lower cognitive reserve, and greater functional limitations [20, 30, 31].

Previous literature has identified orientation difficulties as a risk factor for poor outcomes in older medical patients. For example, a study involving older hospitalized patients found that errors in identifying the date and day of the week were associated with longer LOS. Notably, errors in correctly identifying the year showed 86% sensitivity, 94% specificity, and 37-fold higher odds of having dementia or delirium [30]. Those with impaired orientation also face a higher risk of cognitive decline and conversion from MCI to dementia [32, 33]. Importantly, orientation items on cognitive screening tools have been suggested to be the most predictive for everyday functioning, compared with registration/learning, attention, verbal recall, and language [34]. As orientation engages multiple cognitive domains, it likely acts as a proxy for global cognitive disorganization and functional decline, reflecting broader cognitive vulnerability and a higher risk of adverse outcomes compared to other screening items. In our study involving older surgical patients, its associations with adverse outcomes remained significant after adjusting for preoperative CI status, suggesting that orientation may capture aspects of perioperative vulnerability overlooked by assessing global cognition alone.

It is important to note that these findings are hypothesis-generating as cognitive performance was only examined using a cognitive screening tool, rather than comprehensive neuropsychological batteries [34]. Although the MoCA has been validated in surgical patients, it only includes a limited number of items, which may reduce the reliability and construct validity of domain-specific performance and its sensitivity to capture subtle impairments in specific domains [11]. As preoperative clinics often lack the time, resources, and trained personnel required for administering neuropsychological batteries or even longer screening tools like the MoCA, assessing orientation alone may serve as a simpler first step. Given that “orientation X3” is commonly assessed as part of routine practice by the healthcare team, incorporating “orientation X6” into routine preoperative evaluations likely places minimal burden on patients and perioperative resources. Besides its feasibility within the preoperative setting, our secondary exploratory analysis suggested that orientation may warrant further investigation as a clinically relevant measure to help identify patients who may benefit from closer perioperative monitoring and tailored care.

In addition to orientation, we found that poorer executive function and delayed recall were associated with twofold higher adjusted odds of postoperative delirium, although they were no longer significant after multiplicity adjustment. Previous studies have revealed that impaired memory and executive function were associated with postoperative delirium [13–15, 35]. Given that delirium may be prevented in up to 40% of patients, future studies should investigate whether proactively assessing executive function, memory, and particularly orientation before surgery could help identify patients at risk of delirium and its wide-ranging adverse outcomes [36–38]. Patients identified as being at higher risk for delirium may benefit from targeted risk counselling and mitigation strategies, such as tailored, family-involved Hospital Elder Life Program, educational materials on CI and delirium, prehabilitation interventions, and preoperative nurse-led orientation programs [36, 39–41].

### Limitations

The MoCA domain scores are not equivalent to full neuropsychological measures and may lack psychometric robustness. As the MoCA includes a small number of items per domain, it may have limited sensitivity, reliability, and construct validity for detecting domain-specific impairment. To date, the classifications of MoCA domains remain heterogeneous across literature. Thus, our domain classification and scoring approach followed the original MoCA framework [10, 20]. Due to time and resource constraints, we were unable to administer neuropsychological batteries, limiting our ability to compare performance between these standardized assessments and the MoCA. Nevertheless, the MoCA has good diagnostic accuracy in surgical populations, and its domain scores showed empirical validity when compared with neuropsychological batteries [11, 16]. The MoCA was administered at a single time point, precluding an understanding of whether these deficits were chronic or transient. Accordingly, findings from this exploratory, hypothesis-generating analysis should be interpreted with caution.

To mitigate the influence of education level on MoCA performance and reduce the risk of misclassifying CI, we excluded those with $<$ 8 years of education and adjusted for education level in the total MoCA score as per routine practice in all multivariable analyses. Excluding participants with lower educational attainment may have introduced selection bias toward individuals who differed in cognitive performance, functional status, and sociodemographic backgrounds, potentially underestimating the prevalence of CI. Overall, a substantial proportion of participants were white, highly educated, had significant comorbidities, and underwent orthopedic surgery, limiting the external validity and generalizability of our findings. The low event count of several outcomes limited the number and consistency of covariates that could be included across the multivariable models, potentially leading to residual confounding. Specifically, the low delirium rate in our institutions was likely attributable to the Enhanced Recovery After Surgery protocol, pain management using multimodal non-opioid analgesia, and short LOS [17, 42, 43]. Although additional sensitivity analyses were conducted to address confounding, other potentially relevant variables, such as preoperative medication use, other comorbidities, and intraoperative factors, were not accounted for in the models to avoid statistical overfitting. Given the omission of these variables, our models may not have fully accounted for clinical heterogeneity, potentially influencing the observed associations in our exploratory study. Furthermore, participant responses to certain orientation items such as place and city may be influenced by familiarity with the environment, increasing the risk of false positive findings. In clinical practice, these responses should be interpreted within the context of patients’ environmental familiarity and overall cognitive presentation.

Given the post hoc nature of the analysis, our study may have been insufficiently powered to detect domain-level associations. Although our secondary exploratory analysis demonstrated associations between preoperative orientation scores and adverse outcomes, particularly postoperative delirium, caution is needed when interpreting these results. To avoid missing potentially meaningful associations, our exploratory analytical approach prioritized minimizing type II errors at the risk of inflating type I errors due to multiple testing and equal weighting of adverse outcomes. Multiplicity within and across MoCA domains was considered and addressed in our sensitivity analyses to ensure the overall false discovery rate was not underestimated. Future investigations with a larger sample size should evaluate the distribution of preoperative MoCA scores and their associated characteristics and adverse outcomes in older adults with lower educational attainment and diverse sociodemographic backgrounds undergoing heterogeneous surgical procedures.

## Conclusion

Using the MoCA, this exploratory analysis characterized preoperative cognitive performance in older adults and found that participants screening positive for CI exhibited difficulties across multiple cognitive domains. Specifically, participants scoring $\le$ 25 on the MoCA before surgery performed significantly poorer across all domains of executive/visuospatial function, naming, attention, language, abstraction, delayed recall, and orientation than those scoring > 25. Secondary analyses suggested that only orientation was associated with 30-day adverse outcomes, with lower orientation scores remaining associated with postoperative delirium after more conservative adjustments for confounders and multiplicity within and across MoCA domains. These exploratory findings highlight the importance of characterizing domain-specific cognitive performance and suggest that preoperative orientation difficulties may warrant further investigation in older surgical patients.

## Supplementary Information

Supplementary Material 1.

## Data Availability

Study data are available from the corresponding author upon reasonable request.

## Abbreviations

ASA: American Society of Anesthesiologists
BH: Benjamini-Hochberg
CI: Cognitive impairment
Detect CI: Detection of Cognitive Impairment
EMR: Electronic medical records
ER: Emergency room
MoCA: Montreal Cognitive Assessment
MCI: Mild cognitive impairment
LOS: Length of stay
OR: Odds ratio
OSA: Obstructive sleep apnea
TIA: Transient ischemic attack
95% CI: 95% confidence interval
