# Supplementary material for: Characterizing preoperative domain-specific performance on the Montreal Cognitive Assessment and exploring its associations with adverse outcomes
Source: Anesthesiol Perioper Sci. 2026 Jun 17;4(1):31. doi: 10.1007/s44254-026-00181-2 (PMC13275512; doi:10.1007/s44254-026-00181-2)
Supplement: Supplementary file 1 — Supplementary Material 1. [file 44254_2026_181_MOESM1_ESM.docx]

# **Supplementary Information (Online Resource 1)**

**Characterizing preoperative domain-specific performance on the Montreal Cognitive Assessment and exploring its associations with adverse outcomes**

Ellene Yan, HBSc, Yasmin Alhamdah, MSc, Aparna Saripella, MSc, Eric Cheuk, BSc, Sazzadul Islam, MSc, David He, MD PhD, Keera N. Fishman PhD, Leif Erik Lovblom, PhD, Maria Carmela Tartaglia, MD, David F. Tang-Wai, MD CM, Jean Wong, MD, Frances Chung, MD*

***Address for correspondence:** Frances Chung, MD, FRCPC, Department of Anesthesia and Pain Management, Toronto Western Hospital, University Health Network, University of Toronto, 399 Bathurst Street, Toronto, ON, M5T 2S8, MCL 2-405, Canada

E-mail: [frances.chung@uhn.ca](mailto:frances.chung@uhn.ca); f.chung@utoronto.ca. Phone: 416-670-4253.

Table of Contents

[Supplementary Information (Online Resource 1) 1](#_Toc227610928)

[Supplementary Table S1. STROBE reporting checklist. 3](#_Toc227610929)

[Supplementary Table S2. Performance on individual cognitive domains assessed by the MoCA 5](#_Toc227610930)

[Supplementary Table S3. Correlation matrix of MoCA scores across cognitive domains. 6](#_Toc227610931)

[Supplementary Table S4. Univariable and multivariable analyses of preoperative factors associated with MoCA scores on executive/visuospatial function. 7](#_Toc227610932)

[Supplementary Table S5. Univariable and multivariable analyses of preoperative factors associated with MoCA scores on naming. 9](#_Toc227610933)

[Supplementary Table S6. Univariable and multivariable analyses of preoperative factors associated with MoCA scores on attention. 11](#_Toc227610934)

[Supplementary Table S7. Univariable and multivariable analyses of preoperative factors associated with MoCA scores on language. 13](#_Toc227610935)

[Supplementary Table S8. Univariable and multivariable analyses of preoperative factors associated with MoCA scores on abstraction. 15](#_Toc227610936)

[Supplementary Table S9. Univariable and multivariable analyses of preoperative factors associated with MoCA scores on delayed recall. 16](#_Toc227610937)

[Supplementary Table S10. Univariable and multivariable analyses of preoperative factors associated with MoCA scores on orientation. 17](#_Toc227610938)

[Supplementary Table S11. Preoperative MoCA scores associated with adverse outcomes at 30 and 90 days postoperatively. 18](#_Toc227610939)

[Supplementary Table S12. Associations between poorer MoCA performance on executive/visuospatial function and the incidence of adverse outcomes at 30 and 90 days postoperatively. 22](#_Toc227610940)

[Supplementary Table S13. Associations between poorer MoCA performance on naming and the incidence of adverse outcomes at 30 and 90 days postoperatively. 24](#_Toc227610941)

[Supplementary Table S14. Associations between poorer MoCA performance on attention and the incidence of adverse outcomes at 30 and 90 days postoperatively. 26](#_Toc227610942)

[Supplementary Table S15. Associations between poorer MoCA performance on language and the incidence of adverse outcomes at 30 and 90 days postoperatively. 28](#_Toc227610943)

[Supplementary Table S16. Associations between poorer MoCA performance on abstraction and the incidence of adverse outcomes at 30 and 90 days postoperatively. 30](#_Toc227610944)

[Supplementary Table S17. Associations between poorer MoCA performance on delayed recall and the incidence of adverse outcomes at 30 and 90 days postoperatively. 32](#_Toc227610945)

[Supplementary Table S18. Associations between poorer MoCA performance on orientation and the incidence of adverse outcomes at 30 and 90 days postoperatively. 34](#_Toc227610946)

[Supplementary Table S19. Sensitivity analyses examining the association between poorer MoCA performance and adverse outcomes. 35](#_Toc227610947)

[Supplementary Table S20. Sensitivity analyses examining the association between poorer orientation performance and adverse outcomes. 36](#_Toc227610948)

[Supplementary Table S21. Sensitivity analyses for unmeasured confounding in the associations between poorer orientation performance and adverse postoperative outcomes. 37](#_Toc227610949)

#

# **Supplementary Table S1. STROBE reporting checklist.**

|  | Item No | Recommendation | Page No |
| --- | --- | --- | --- |
| **Title and abstract** | 1 | (*a*) Indicate the study’s design with a commonly used term in the title or the abstract | 1-2 |
|  |  | (*b*) Provide in the abstract an informative and balanced summary of what was done and what was found |  |
| Introduction | | | |
| Background/rationale | 2 | Explain the scientific background and rationale for the investigation being reported | 4 |
| Objectives | 3 | State specific objectives, including any prespecified hypotheses | 4-5 |
| Methods | | | |
| Study design | 4 | Present key elements of study design early in the paper | 5 |
| Setting | 5 | Describe the setting, locations, and relevant dates, including periods of recruitment, exposure, follow-up, and data collection | 5 |
| Participants | 6 | Give the eligibility criteria, and the sources and methods of selection of participants. Describe methods of follow-up | 5 |
|  |  |  |  |
| Variables | 7 | Clearly define all outcomes, exposures, predictors, potential confounders, and effect modifiers. Give diagnostic criteria, if applicable | 5-7 |
| Data sources/ measurement | 8* | For each variable of interest, give sources of data and details of methods of assessment (measurement). Describe comparability of assessment methods if there is more than one group | 5-7 |
| Bias | 9 | Describe any efforts to address potential sources of bias | 7-8 |
| Study size | 10 | Explain how the study size was arrived at | 8 |
| Quantitative variables | 11 | Explain how quantitative variables were handled in the analyses. If applicable, describe which groupings were chosen and why | 7-8 |
| Statistical methods | 12 | (*a*) Describe all statistical methods, including those used to control for confounding | 7-9 |
|  |  | (*b*) Describe any methods used to examine subgroups and interactions |  |
|  |  | (*c*) Explain how missing data were addressed |  |
|  |  | (*d*) If applicable, explain how loss to follow-up was addressed |  |
|  |  | (*e*) Describe any sensitivity analyses |  |
| Results | | |  |
| Participants | 13* | (a) Report numbers of individuals at each stage of study—eg numbers potentially eligible, examined for eligibility, confirmed eligible, included in the study, completing follow-up, and analysed | 9  Fig. 1 |
|  |  | (b) Give reasons for non-participation at each stage |  |
|  |  | (c) Consider use of a flow diagram |  |
| Descriptive data | 14* | (a) Give characteristics of study participants (eg demographic, clinical, social) and information on exposures and potential confounders | 9  Table 1 |
|  |  | (b) Indicate number of participants with missing data for each variable of interest |  |
|  |  | (c) Summarise follow-up time (eg, average and total amount) |  |
| Outcome data | 15* | Report numbers of outcome events or summary measures over time | Supplementary Tables S2, S11 |

| Main results | 16 | (*a*) Give unadjusted estimates and, if applicable, confounder-adjusted estimates and their precision (eg, 95% confidence interval). Make clear which confounders were adjusted for and why they were included | 9-13  Tables 2-3  Fig. 2  Supplementary Tables S3-10; S12-18 |
| --- | --- | --- | --- |
|  |  | (*b*) Report category boundaries when continuous variables were categorized |  |
|  |  | (*c*) If relevant, consider translating estimates of relative risk into absolute risk for a meaningful time period |  |
| Other analyses | 17 | Report other analyses done—eg analyses of subgroups and interactions, and sensitivity analyses | 12-  Supplementary Tables S12-21 |
| Discussion | | | |
| Key results | 18 | Summarise key results with reference to study objectives | 13-16 |
| Limitations | 19 | Discuss limitations of the study, taking into account sources of potential bias or imprecision. Discuss both direction and magnitude of any potential bias | 16-17 |
| Interpretation | 20 | Give a cautious overall interpretation of results considering objectives, limitations, multiplicity of analyses, results from similar studies, and other relevant evidence | 13-17 |
| Generalisability | 21 | Discuss the generalisability (external validity) of the study results | 16-17 |
| Other information | | | |
| Funding | 22 | Give the source of funding and the role of the funders for the present study and, if applicable, for the original study on which the present article is based | 19 |

# **Supplementary Table S2. Performance on individual cognitive domains assessed by the MoCA**

| **Cognitive Domain**  **(Possible maximum score)** | **Total**  **(n=382)** | **No-CI**  **(n=247)** | **CI***  **(n=135)** | **P-value** |
| --- | --- | --- | --- | --- |
| **1. Executive/Visuospatial Function (5)** | **3.86 ± 1.03** | **4.22 ± 0.85** | **3.21 ± 1.02** | $\mathbf{<}$**0.001** |
|  | **4 [3, 5]** | **4 [4, 5]** | **3 [3, 4]** | $\mathbf{<}$**0.001** |
| 1. **Trail Making Test (1)** | **0.71 ± 0.45** | **0.84 ± 0.37** | **0.49 ± 0.50** | $\mathbf{<}$**0.001** |
|  | **1 [0, 1]** | **1 [1, 1]** | **0 [0, 1]** | $\mathbf{<}$**0.001** |
| 1. **Cube Copy (1)** | **0.66 ± 0.47** | **0.76 ± 0.43** | **0.49 ± 0.50** | $\mathbf{<}$**0.001** |
|  | **1 [0, 1]** | **1 [1, 1]** | **0 [0, 1]** | $\mathbf{<}$**0.001** |
| 1. **Clock Drawing (3)** | **2.49 ± 0.64** | **2.62 ± 0.55** | **2.24 ± 0.73** | $\mathbf{<}$**0.001** |
|  | **3 [2, 3]** | **3 [2, 3]** | **2 [2, 3]** | $\mathbf{<}$**0.001** |
| **2. Naming (3)** | **2.90 ± 0.33** | **2.95 ± 0.22** | **2.81 ± 0.46** | **0.001** |
|  | **3 [3, 3]** | **3 [3, 3]** | **3 [3, 3]** | $\mathbf{<}$**0.001** |
| **3. Attention (6)** | **5.59 ± 0.80** | **5.86 ± 0.42** | **5.11 ± 1.06** | $\mathbf{<}$**0.001** |
|  | **6 [5, 6]** | **6 [6, 6]** | **5 [4, 6]** | $\mathbf{<}$**0.001** |
| 1. **Forward & Backward Digit Span (2)** | **1.87 ± 0.37** | **1.94 ± 0.26** | **1.74 ± 1.49** | $\mathbf{<}$**0.001** |
|  | **2 [2, 2]** | **2 [2, 2]** | **2 [2, 2]** | $\mathbf{<}$**0.001** |
| 1. **Vigilance (1)** | **0.98 ± 0.13** | **0.99 ± 0.09** | **0.96 ± 0.19** | **0.10** |
|  | **1 [1, 1]** | **1 [1, 1]** | **1 [1, 1]** | **0.044** |
| 1. **Serial 7 Subtraction (3)** | **2.74 ± 0.63** | **2.93 ± 0.33** | **2.41 ± 0.88** | $\mathbf{<}$**0.001** |
|  | **3 [3, 3]** | **3 [3, 3]** | **3 [2, 3]** | $\mathbf{<}$**0.001** |
| **4. Language (3)** | **2.48 ± 0.76** | **2.70 ± 0.50** | **2.07 ± 0.96** | $\mathbf{<}$**0.001** |
|  | **3 [2, 3]** | **3 [2, 3]** | **2 [1, 3]** | $\mathbf{<}$**0.001** |
| 1. **Sentence Repetition (2)** | **1.61 ± 0.62** | **1.76 ± 0.46** | **1.34 ± 0.77** | $\mathbf{<}$**0.001** |
|  | **2 [1, 2]** | **2 [2, 2]** | **2 [1, 2]** | $\mathbf{<}$**0.001** |
| 1. **Verbal Fluency (1)** | **0.87 ± 0.34** | **0.94 ± 0.23** | **0.73 ± 0.45** | $\mathbf{<}$**0.001** |
|  | **1 [1, 1]** | **1 [1, 1]** | **1 [0, 1]** | $\mathbf{<}$**0.001** |
| **5. Abstraction (2)** | **1.88 ± 0.36** | **1.95 ± 0.22** | **1.77 ± 0.50** | $\mathbf{<}$**0.001** |
|  | **2 [2, 2]** | **2 [2, 2]** | **2 [2, 2]** | $\mathbf{<}$**0.001** |
| **6. Delayed Recall (5)** | **3.14 ± 1.48** | **3.81 ± 1.07** | **1.90 ± 1.33** | $\mathbf{<}$**0.001** |
|  | **3 [2, 4]** | **4 [3, 5]** | **2 [1, 3]** | $\mathbf{<}$**0.001** |
| **7. Orientation (6)** | **5.96 ± 0.23** | **5.98 ± 0.13** | **5.90 ± 0.34** | **0.010** |
|  | **6 [6, 6]** | **6 [6, 6]** | **6 [6, 6]** | **0.002** |

Values expressed as mean ± standard deviation and median [interquartile range]. Scores range from 0-5 for executive function, 0-3 for naming, 0-6 for attention, 0-3 for language, 0-2 for abstraction, 0-5 for delayed recall, and 0-6 for orientation (lower scores indicate poorer cognitive performance). Abbreviation: CI, cognitive impairment. *Classification of CI and No-CI was based on the established MoCA cut-off of $\leq$25. P-values of $\leq$0.05 indicate statistical significance.

# **Supplementary Table S3. Correlation matrix of MoCA scores across cognitive domains.**

|  | **Naming** | **Attention** | **Language** | **Abstraction** | **Delayed Recall** | **Orientation** |
| --- | --- | --- | --- | --- | --- | --- |
| **Executive/ visuospatial function** | **0.07** | **0.21**** | **0.19**** | **0.13*** | **0.13*** | **0.11*** |
| **Naming** | **-** | **0.10** | **0.10** | **0.09** | **0.09** | **-0.01** |
| **Attention** | **-** | **-** | **0.10*** | **0.19**** | **0.20**** | **0.01** |
| **Language** | **-** | **-** | **-** | **0.15*** | **0.12*** | **0.00** |
| **Abstraction** | **-** | **-** | **-** | **-** | **0.13*** | **0.16*** |
| **Delayed Recall** | **-** | **-** | **-** | **-** | **-** | **0.10*** |

Values expressed as Spearman's rho (ρ). **P-values of $\leq$0.001, * P-values of $\leq$0.05 between cognitive domains.

# **Supplementary Table S4. Univariable and multivariable analyses of preoperative factors associated with MoCA scores on executive/visuospatial function.**

| **Baseline Characteristic** | **Comparison** | **Univariable Analysis** | | **Multivariable Analysis^a^** | |
| --- | --- | --- | --- | --- | --- |
|  |  | **Unadjusted estimate**  **(95% CI)** | **Unadjusted P-value** | **Adjusted estimate (95% CI)** | **Adjusted P-value** |
| **Age** | **+5 years** | **-0.11 (-0.21, -0.02)** | **0.016** | **-0.094 (-0.188, 0.001)** | **0.052** |
| **Sex** | **Female vs Male** | **-0.13 (-0.34, 0.08)** | **0.23** | **-0.12 (-0.34, 0.10)** | **0.28** |
| **Education** | **12 years vs** $\mathbf{>}$**12** | **-0.46 (-0.77, -0.16)** | **0.003** | **-0.34 (-0.65, -0.02)** | **0.037** |
| **Race/ethnicity** | **White vs Other** | **0.34 (0.01, 0.66)** | **0.041** | **-** | **-** |
| **ASA** | **III-IV vs I-II** | **-0.27 (-0.58, 0.04)** | **0.08** | **-0.23 (-0.55, 0.08)** | **0.15** |
| **CAD** | **Yes vs No** | **-0.23 (-0.57, 0.12)** | **0.19** | **-** | **-** |
| **Hypertension** | **Yes vs No** | **-0.17 (-0.38, 0.03)** | **0.10** | **-** | **-** |
| **TIA/Stroke** | **Yes vs No** | **-0.52 (-0.99, -0.05)** | **0.031** | **-0.48 (-0.96, 0.01)** | **0.055** |
| **OSA** | **Yes vs No** | **0.12 (-0.13, 0.38)** | **0.34** | **0.17 (-0.09, 0.43)** | **0.20** |
| **Frailty** | **Yes vs No** | **-0.14 (-0.43, 0.16)** | **0.36** | **0.03 (-0.30, 0.35)** | **0.87** |
| **Clinically significant disability (WHODAS 35%)** | **Disability vs No Disability** | **-0.32 (-0.61, -0.03)** | **0.030** | **-0.24 (-0.57, 0.10)** | **0.17** |
| **Anxiety/Depression (PHQ3)** | **Presence vs Absence of anxiety/depression (any severity)** | **0.02 (-0.21, 0.25)** | **0.88** | **0.11 (-0.14, 0.37)** | **0.38** |
| **Pain (VAS: 1-10)** | **+1 (higher pain)** | **-0.05 (-0.09, -0.02)** | **0.002** | **-** | **-** |
| **SQS (VAS: 1-10)** | **-1 (poorer sleep)** | **0.01 (-0.03, 0.05)** | **0.65** | **0.006 (-0.04, 0.05)** | **0.77** |

Abbreviations: ASA, American Society of Anesthesiologists; CAD, coronary artery disease; OSA, obstructive sleep apnea; PHQ, Patient Health Questionnaire; SQS, Single-item Sleep Quality Scale; TIA, Transient ischemic attack; VAS, visual analog scale, WHODAS, World Health Organization Disability Assessment Schedule. Estimates expressed as beta-coefficients for continuous outcomes. Total MoCA scores ranged from 0-30, with lower scores indicating poorer cognitive performance. ^a^Multivariable analyses adjusted for age, sex, education, American Society of Anesthesiologists (ASA) physical status, transient ischemic attack (TIA)/stroke, obstructive sleep apnea (OSA), frailty, clinically significant functional disability, anxiety/depression, and sleep quality. Race/ethnicity, coronary artery disease (CAD), hypertension, and pain were not included to avoid statistical overfitting. P-values of $\leq$0.05 indicate statistical significance.

# **Supplementary Table S5. Univariable and multivariable analyses of preoperative factors associated with MoCA scores on naming.**

| **Baseline Characteristic** | **Comparison** | **Univariable Analysis** | | **Multivariable Analysis^a^** | |
| --- | --- | --- | --- | --- | --- |
|  |  | **Unadjusted estimate**  **(95% CI)** | **Unadjusted P-value** | **Adjusted estimate (95% CI)** | **Adjusted P-value** |
| **Age** | **+5 years** | **-0.001 (-0.031, 0.029)** | **0.94** | **-0.002 (-0.032, 0.027)** | **0.88** |
| **Sex** | **Female vs male** | **-0.02 (-0.08, 0.05)** | **0.62** | **-0.01 (-0.08, 0.06)** | **0.78** |
| **Education** | **12 years vs** $\mathbf{>}$**12** | **-0.23 (-0.33, -0.14)** | $\mathbf{<}$**0.001** | **-0.20 (-0.30, -0.10)** | $\mathbf{<}$**0.001** |
| **Race/ethnicity** | **White vs other** | **0.20 (0.10, 0.30)** | $\mathbf{<}$**0.001** | **-** | **-** |
| **ASA** | **III-IV vs I-II** | **-0.04 (-0.14, 0.06)** | **0.42** | **-0.03 ( -0.13, 0.07)** | **0.54** |
| **CAD** | **Yes vs no** | **-0.07 (-0.18, 0.04)** | **0.23** | **-** | **-** |
| **Hypertension** | **Yes vs no** | **-0.02 (-0.09, 0.05)** | **0.56** | **-** | **-** |
| **TIA/Stroke** | **Yes vs no** | **-0.06 (-0.22, 0.09)** | **0.41** | **-0.06 (-0.22, 0.09)** | **0.40** |
| **OSA** | **Yes vs no** | **-0.04 (-0.12, 0.05)** | **0.39** | **0.01 (-0.07, 0.09)** | **0.85** |
| **Frailty** | **Yes vs no** | **-0.04 (-0.13, 0.05)** | **0.41** | **-0.01 (-0.11, 0.09)** | **0.83** |
| **Clinically significant disability (WHODAS 35%)** | **Disability vs No Disability** | **-0.06 (-0.15, 0.03)** | **0.22** | **-0.02 (-0.12, 0.09)** | **0.75** |
| **Anxiety/Depression (PHQ3)** | **Presence vs absence of anxiety/depression (any severity)** | **-0.002 (-0.075, 0.071)** | **0.95** | **0.02 (-0.06, 0.10)** | **0.59** |
| **Pain (VAS: 1-10)** | **+1 (higher pain)** | **-0.008 (-0.018, 0.003)** | **0.15** | **-** | **-** |
| **SQS (VAS: 1-10)** | **-1 (poorer sleep)** | **-0.01 (-0.02, 0.01)** | **0.37** | **-0.005 (-0.018, 0.009)** | **0.49** |

Abbreviations: ASA, American Society of Anesthesiologists; CAD, coronary artery disease; OSA, obstructive sleep apnea; PHQ, Patient Health Questionnaire; SQS, Single-item Sleep Quality Scale; TIA, Transient ischemic attack; VAS, visual analog scale, WHODAS, World Health Organization Disability Assessment Schedule. Estimates expressed as beta-coefficients for continuous outcomes. Total MoCA scores ranged from 0-30, with a lower score indicating poorer cognitive performance. ^a^Multivariable analyses adjusted for age, sex, education, American Society of Anesthesiologists (ASA) physical status, transient ischemic attack (TIA)/stroke, obstructive sleep apnea (OSA), frailty, clinically significant functional disability, anxiety/depression, and sleep quality. Race/ethnicity, coronary artery disease (CAD), hypertension, and pain were not included to avoid statistical overfitting. P-values of $\leq$0.05 indicate statistical significance.

# **Supplementary Table S6. Univariable and multivariable analyses of preoperative factors associated with MoCA scores on attention.**

| **Baseline Characteristic** | **Comparison** | **Univariable Analysis** | | **Multivariable Analysis^a^** | |
| --- | --- | --- | --- | --- | --- |
|  |  | **Unadjusted estimate**  **(95% CI)** | **Unadjusted P-value** | **Adjusted estimate (95% CI)** | **Adjusted P-value** |
| **Age** | **+5 years** | **-0.03 (-0.10, 0.04)** | **0.43** | **-0.03 (-0.10, 0.04)** | **0.40** |
| **Sex** | **Female vs male** | **-0.08 (-0.24, 0.08)** | **0.34** | **-0.08 (-0.25, 0.09)** | **0.35** |
| **Education** | **12 years vs** $\mathbf{>}$**12** | **-0.20 (-0.44, 0.04)** | **0.10** | **-0.21 (-0.46, 0.03)** | **0.08** |
| **Race/ethnicity** | **White vs other** | **0.19 (-0.05, 0.44)** | **0.12** | **-** | **-** |
| **ASA** | **III-IV vs I-II** | **0.003 (-0.237, 0.243)** | **0.98** | **-0.02 (-0.26, 0.23)** | **0.90** |
| **CAD** | **Yes vs no** | **0.13 (-0.14, 0.40)** | **0.34** | **-** | **-** |
| **Hypertension** | **Yes vs no** | **-0.09 (-0.25, 0.07)** | **0.27** | **-** | **-** |
| **TIA/Stroke** | **Yes vs no** | **-0.57 (-0.93, -0.21)** | **0.002** | **-0.53 (-0.91, -0.16)** | **0.005** |
| **OSA** | **Yes vs no** | **0.12 (-0.08, 0.31)** | **0.24** | **0.20 (0.001, 0.402)** | **0.053** |
| **Frailty** | **Yes vs no** | **-0.04 (-0.27, 0.18)** | **0.71** | **0.04 (-0.21, 0.30)** | **0.73** |
| **Clinically significant disability (WHODAS 35%)** | **Disability vs No Disability** | **-0.14 (-0.36, 0.08)** | **0.22** | **-0.09 (-0.35, 0.17)** | **0.49** |
| **Anxiety/Depression (PHQ3)** | **Presence vs absence of anxiety/depression (any severity)** | **-0.003 (-0.182, 0.176)** | **0.97** | **0.07 (-0.13, 0.26)** | **0.51** |
| **Pain (VAS: 1-10)** | **+1 (higher pain)** | **-0.02 (-0.04, 0.01)** | **0.17** | **-** | **-** |
| **SQS (VAS: 1-10)** | **-1 (poorer sleep)** | **-0.01 (-0.04, 0.02)** | **0.56** | **-0.01 (-0.05, 0.02)** | **0.42** |

Abbreviations: ASA, American Society of Anesthesiologists; CAD, coronary artery disease; OSA, obstructive sleep apnea; PHQ, Patient Health Questionnaire; SQS, Single-item Sleep Quality Scale; TIA, Transient ischemic attack; VAS, visual analog scale, WHODAS, World Health Organization Disability Assessment Schedule. Estimates expressed as beta-coefficients for continuous outcomes. Total MoCA scores ranged from 0-30, with a lower score indicating poorer cognitive performance. ^a^Multivariable analyses adjusted for age, sex, education, American Society of Anesthesiologists (ASA) physical status, transient ischemic attack (TIA)/stroke, obstructive sleep apnea (OSA), frailty, clinically significant functional disability, anxiety/depression, and sleep quality. Race/ethnicity, coronary artery disease (CAD), hypertension, and pain were not included to avoid statistical overfitting. P-values of $\leq$0.05 indicate statistical significance.

# **Supplementary Table S7. Univariable and multivariable analyses of preoperative factors associated with MoCA scores on language.**

| **Baseline Characteristic** | **Comparison** | **Univariable Analysis** | | **Multivariable Analysis^a^** | |
| --- | --- | --- | --- | --- | --- |
|  |  | **Unadjusted estimate**  **(95% CI)** | **Unadjusted P-value** | **Adjusted estimate (95% CI)** | **Adjusted P-value** |
| **Age** | **+5 years** | **-0.02 (-0.09, 0.05)** | **0.59** | **-0.01 (-0.08, 0.06)** | **0.77** |
| **Sex** | **Female vs male** | **0.03 (-0.13, 0.18)** | **0.71** | **0.04 (-0.12, 0.20)** | **0.60** |
| **Education** | **12 years vs** $\mathbf{>}$**12** | **-0.43 (-0.66, -0.21)** | $\mathbf{<}$**0.001** | **-0.43 (-0.67, -0.20)** | $\mathbf{<}$**0.001** |
| **Race/ethnicity** | **White vs other** | **0.46 (0.23, 0.70)** | $\mathbf{<}$**0.001** | **-** | **-** |
| **ASA** | **III-IV vs I-II** | **-0.11 (-0.34, 0.12)** | **0.35** | **-0.15 (-0.38, 0.09)** | **0.22** |
| **CAD** | **Yes vs no** | **-0.03 (-0.29, 0.22)** | **0.81** | **-** | **-** |
| **Hypertension** | **Yes vs no** | **-0.07 (-0.23, 0.08)** | **0.35** | **-** | **-** |
| **TIA/Stroke** | **Yes vs no** | **0.05 (-0.30, 0.41)** | **0.77** | **0.04 (-0.32, 0.40)** | **0.83** |
| **OSA** | **Yes vs no** | **0.1879 (0.0002, 0.3756)** | **0.0498** | **0.27 (0.08, 0.47)** | **0.005** |
| **Frailty** | **Yes vs no** | **0.08 (-0.14, 0.29)** | **0.50** | **0.17 (-0.07, 0.41)** | **0.16** |
| **Clinically significant disability (WHODAS 35%)** | **Disability vs No Disability** | **-0.21 (-0.42, 0.01)** | **0.06** | **-0.26 (-0.51, -0.01)** | **0.041** |
| **Anxiety/Depression (PHQ3)** | **Presence vs absence of anxiety/depression (any severity)** | **0.07 (-0.10, 0.24)** | **0.44** | **0.10 (-0.08, 0.29)** | **0.27** |
| **Pain (VAS: 1-10)** | **+1 (higher pain)** | **0.01 (-0.02, 0.03)** | **0.58** | **-** | **-** |
| **SQS (VAS: 1-10)** | **-1 (poorer sleep)** | **0.005 (-0.027, 0.036)** | **0.77** | **0.0005 (-0.0311, 0.0320)** | **0.98** |

Abbreviations: ASA, American Society of Anesthesiologists; CAD, coronary artery disease; OSA, obstructive sleep apnea; PHQ, Patient Health Questionnaire; SQS, Single-item Sleep Quality Scale; TIA, Transient ischemic attack; VAS, visual analog scale, WHODAS, World Health Organization Disability Assessment Schedule. Estimates expressed as beta-coefficients for continuous outcomes. Total MoCA scores ranged from 0-30, with a lower score indicating poorer cognitive performance. ^a^Multivariable analyses adjusted for age, sex, education, American Society of Anesthesiologists (ASA) physical status, transient ischemic attack (TIA)/stroke, obstructive sleep apnea (OSA), frailty, clinically significant functional disability, anxiety/depression, and sleep quality. Race/ethnicity, coronary artery disease (CAD), hypertension, and pain were not included to avoid statistical overfitting. P-values of $\leq$0.05 indicate statistical significance.

# **Supplementary Table S8. Univariable and multivariable analyses of preoperative factors associated with MoCA scores on abstraction.**

| **Baseline Characteristic** | **Comparison** | **Univariable Analysis** | | **Multivariable Analysis^a^** | |
| --- | --- | --- | --- | --- | --- |
|  |  | **Unadjusted estimate**  **(95% CI)** | **Unadjusted P-value** | **Adjusted estimate (95% CI)** | **Adjusted P-value** |
| **Age** | **+5 years** | **-0.02 (-0.05, 0.02)** | **0.34** | **-0.02 (-0.05, 0.01)** | **0.27** |
| **Sex** | **Female vs male** | **-0.06 (-0.13, 0.01)** | **0.11** | **-0.06 (-0.13, 0.014)** | **0.11** |
| **Education** | **12 years vs** $\mathbf{>}$**12** | **-0.24 (-0.34, -0.13)** | $\mathbf{<}$**0.001** | **-0.18 (-0.28, -0.07)** | **0.001** |
| **Race/ethnicity** | **White vs other** | **0.18 (0.07, 0.29)** | **0.002** | **-** | **-** |
| **ASA** | **III-IV vs I-II** | **0.01 (-0.10, 0.12)** | **0.88** | **0.03 (-0.07, 0.14)** | **0.54** |
| **CAD** | **Yes vs no** | **-0.08 (-0.20, 0.04)** | **0.21** | **-** | **-** |
| **Hypertension** | **Yes vs no** | **-0.01 (-0.08, 0.06)** | **0.80** | **-** | **-** |
| **TIA/Stroke** | **Yes vs no** | **-0.10 (-0.27, 0.07)** | **0.24** | **-0.11 (-0.27, 0.05)** | **0.17** |
| **OSA** | **Yes vs no** | **-0.03 (-0.12, 0.06)** | **0.53** | **0.01 (-0.08, 0.10)** | **0.82** |
| **Frailty** | **Yes vs no** | **-0.05 (-0.15, 0.05)** | **0.35** | **0.02 (-0.09, 0.13)** | **0.75** |
| **Clinically significant disability (WHODAS 35%)** | **Disability vs No Disability** | **-0.13 (-0.22, -0.03)** | **0.009** | **-0.115 (-0.227, -0.003)** | **0.044** |
| **Anxiety/Depression (PHQ3)** | **Presence vs absence of anxiety/depression (any severity)** | **0.01 (-0.06, 0.09)** | **0.74** | **0.06 (-0.03, 0.14)** | **0.18** |
| **Pain (VAS: 1-10)** | **+1 (higher pain)** | **-0.007 (-0.019, 0.004)** | **0.21** | **-** | **-** |
| **SQS (VAS: 1-10)** | **-1 (poorer sleep)** | **-0.003 (-0.017, 0.011)** | **0.70** | **-0.002 (-0.016, 0.012)** | **0.75** |

Abbreviations: ASA, American Society of Anesthesiologists; CAD, coronary artery disease; OSA, obstructive sleep apnea; PHQ, Patient Health Questionnaire; SQS, Single-item Sleep Quality Scale; TIA, Transient ischemic attack; VAS, visual analog scale, WHODAS, World Health Organization Disability Assessment Schedule. Estimates expressed as beta-coefficients for continuous outcomes. Total MoCA scores ranged from 0-30, with a lower score indicating poorer cognitive performance. ^a^Multivariable analyses adjusted for age, sex, education, American Society of Anesthesiologists (ASA) physical status, transient ischemic attack (TIA)/stroke, obstructive sleep apnea (OSA), frailty, clinically significant functional disability, anxiety/depression, and sleep quality. Race/ethnicity, coronary artery disease (CAD), hypertension, and pain were not included to avoid statistical overfitting. P-values of $\leq$0.05 indicate statistical significance.

# **Supplementary Table S9. Univariable and multivariable analyses of preoperative factors associated with MoCA scores on delayed recall.**

| **Baseline Characteristic** | **Comparison** | **Univariable Analysis** | | **Multivariable Analysis^a^** | |
| --- | --- | --- | --- | --- | --- |
|  |  | **Unadjusted estimate**  **(95% CI)** | **Unadjusted P-value** | **Adjusted estimate (95% CI)** | **Adjusted P-value** |
| **Age** | **+5 years** | **-0.17 (-0.30, -0.04)** | **0.013** | **-0.17 (-0.31, -0.03)** | **0.015** |
| **Sex** | **Female vs male** | **0.40 (0.10, 0.70)** | **0.010** | **0.44 (0.14, 0.76)** | **0.005** |
| **Education** | **12 years vs** $\mathbf{>}$**12** | **-0.55 (-0.99, -0.11)** | **0.015** | **-0.54 (-0.99, -0.08)** | **0.022** |
| **Race/ethnicity** | **White vs other** | **0.05 (-0.42, 0.52)** | **0.84** | **-** | **-** |
| **ASA** | **III-IV vs I-II** | **-0.17 (-0.62, 0.27)** | **0.45** | **-0.05 (-0.51, 0.40)** | **0.81** |
| **CAD** | **Yes vs no** | **-0.24 (-0.74, 0.26)** | **0.35** | **-** | **-** |
| **Hypertension** | **Yes vs no** | **-0.31 (-0.61, -0.01)** | **0.045** | **-** | **-** |
| **TIA/Stroke** | **Yes vs no** | **-0.53 (-1.21, 0.15)** | **0.13** | **-0.42 (-1.11, 0.29)** | **0.25** |
| **OSA** | **Yes vs no** | **0.05 (-0.32, 0.42)** | **0.79** | **0.17 (-0.23, 0.553)** | **0.45** |
| **Frailty** | **Yes vs no** | **-0.09 (-0.51, 0.34)** | **0.69** | **-0.08 (-0.55, 0.40)** | **0.76** |
| **Clinically significant disability (WHODAS 35%)** | **Disability vs No Disability** | **-0.13 (-0.55, 0.29)** | **0.55** | **0.001 (-0.488, 0.490)** | **>0.99** |
| **Anxiety/Depression (PHQ3)** | **Presence vs absence of anxiety/depression (any severity)** | **-0.04 (-0.387, 0.30)** | **0.84** | **-0.12 (-0.48, 0.25)** | **0.54** |
| **Pain (VAS: 1-10)** | **+1 (higher pain)** | **0.01 (-0.04, 0.05)** | **0.83** | **-** | **-** |
| **SQS (VAS: 1-10)** | **-1 (poorer sleep)** | **0.01 (-0.05, 0.07)** | **0.81** | **0.01 (-0.06, 0.07)** | **0.88** |

Abbreviations: ASA, American Society of Anesthesiologists; CAD, coronary artery disease; OSA, obstructive sleep apnea; PHQ, Patient Health Questionnaire; SQS, Single-item Sleep Quality Scale; TIA, Transient ischemic attack; VAS, visual analog scale, WHODAS, World Health Organization Disability Assessment Schedule. Estimates expressed as beta-coefficients for continuous outcomes. Total MoCA scores ranged from 0-30, with a lower score indicating poorer cognitive performance. ^a^Multivariable analyses adjusted for age, sex, education, American Society of Anesthesiologists (ASA) physical status, transient ischemic attack (TIA)/stroke, obstructive sleep apnea (OSA), frailty, clinically significant functional disability, anxiety/depression, and sleep quality. Race/ethnicity, coronary artery disease (CAD), hypertension, and pain were not included to avoid statistical overfitting. P-values of $\leq$0.05 indicate statistical significance.

# **Supplementary Table S10. Univariable and multivariable analyses of preoperative factors associated with MoCA scores on orientation.**

| **Baseline Characteristic** | **Comparison** | **Univariable Analysis** | | **Multivariable Analysis^a^** | |
| --- | --- | --- | --- | --- | --- |
|  |  | **Unadjusted estimate**  **(95% CI)** | **Unadjusted P-value** | **Adjusted estimate (95% CI)** | **Adjusted P-value** |
| **Age** | **+5 years** | **-0.03 (-0.05, -0.01)** | **0.009** | **-0.03 (-0.05, -0.01)** | **0.008** |
| **Sex** | **Female vs male** | **-0.01 (-0.06, 0.03)** | **0.60** | **-0.02 (-0.07, 0.02)** | **0.32** |
| **Education** | **12 years vs** $\mathbf{>}$**12** | **-0.02 (-0.09, 0.05)** | **0.61** | **0.0004 (-0.0696, 0.0704)** | **0.99** |
| **Race** | **White vs other** | **-0.02 (-0.10, 0.05)** | **0.50** | **-** | **-** |
| **ASA** | **III-IV vs I-II** | **-0.004 (-0.074, 0.065)** | **0.91** | **-0.002 (-0.072, 0.068)** | **0.95** |
| **CAD** | **Yes vs no** | **-0.04 (-0.12, 0.04)** | **0.33** | **-** | **-** |
| **Hypertension** | **Yes vs no** | **0.03 (-0.02, 0.08)** | **0.23** | **-** | **-** |
| **TIA/Stroke** | **Yes vs no** | **-0.01 (-0.12, 0.10)** | **0.88** | **-0.02 (-0.13, 0.08)** | **0.67** |
| **OSA** | **Yes vs no** | **-0.01 (-0.06, 0.05)** | **0.81** | **-0.01 (-0.07, 0.05)** | **0.81** |
| **Frailty** | **Yes vs no** | **0.03 (-0.04, 0.09)** | **0.42** | **0.03 (-0.04, 0.10)** | **0.42** |
| **Clinically significant disability (WHODAS 35%)** | **Disability vs No Disability** | **0.01 (-0.06, 0.07)** | **0.86** | **-0.01 (-0.08, 0.07)** | **0.84** |
| **Anxiety/Depression (PHQ3)** | **Presence vs absence of anxiety/depression (any severity)** | **0.03 (-0.02, 0.08)** | **0.25** | **0.02 (-0.04, 0.08)** | **0.50** |
| **Pain (VAS: 1-10)** | **+1 (higher pain)** | **0.003 (-0.004, 0.010)** | **0.42** | **-** | **-** |
| **SQS (VAS: 1-10)** | **-1 (poorer sleep)** | **0.008 (-0.001, 0.017)** | **0.08** | **0.006 (-0.003, 0.016)** | **0.20** |

Abbreviations: ASA, American Society of Anesthesiologists; CAD, coronary artery disease; OSA, obstructive sleep apnea; PHQ, Patient Health Questionnaire; SQS, Single-item Sleep Quality Scale; TIA, Transient ischemic attack; VAS, visual analog scale, WHODAS, World Health Organization Disability Assessment Schedule. Estimates expressed as beta-coefficients for continuous outcomes. Total MoCA scores ranged from 0-30, with a lower score indicating poorer cognitive performance. ^a^Multivariable analyses adjusted for age, sex, education, American Society of Anesthesiologists (ASA) physical status, transient ischemic attack (TIA)/stroke, obstructive sleep apnea (OSA), frailty, clinically significant functional disability, anxiety/depression, and sleep quality. Race/ethnicity, coronary artery disease (CAD), hypertension, and pain were not included to avoid statistical overfitting. P-values of $\leq$0.05 indicate statistical significance.

# **Supplementary Table S11. Preoperative MoCA scores associated with adverse outcomes at 30 and 90 days postoperatively.**

| **Executive function** | | | | |  |
| --- | --- | --- | --- | --- | --- |
| **Clinical outcome** | **With outcome** | | **Without outcome** | |  |
|  | **n (%)** | **Mean ± SD** | **n (%)** | **Mean ± SD** |  |
| **30 days postoperatively** | | | | |  |
| **Postoperative delirium** | **6 (1.6)** | **3.00 ± 1.90** | **376 (98.4)** | **3.88 ± 1.01** |  |
| **All-cause complications** | **123 (32.2)** | **3.81 ± 1.09** | **259 (67.8)** | **3.89 ± 1.00** |  |
| **Non-home discharge** | **46 (12.1)** | **3.94 ± 1.04** | **335 (87.9)** | **3.86 ± 1.03** |  |
| **ER visits** | **42 (11.0)** | **3.83 ± 0.94** | **339 (89.0)** | **3.87 ± 1.04** |  |
| **30-day readmission** | **10 (2.6)** | **3.90 ± 1.10** | **371 (97.4)** | **3.87 ± 1.03** |  |
| **Mortality** | **2 (0.5)** | **3.50 ± 0.71** | **380 (99.5)** | **3.87 ± 1.03** |  |
| **Composite outcomes** | **146 (38.2)** | **3.82 ± 1.09** | **236 (61.8)** | **3.89 ± 0.99** |  |
| **90 days postoperatively** | | | | |  |
| **All-cause complications** | **20 (5.3)** | **4.00 ± 0.92** | **359 (94.7)** | **3.86 ± 1.03** |  |
| **ER visits** | **14 (3.7)** | **4.36 ± 0.75** | **365 (96.3)** | **3.85 ± 1.03** |  |
| **Mortality** | **2 (0.5)** | **3.50 ± 2.12** | **377 (99.5)** | **3.87 ± 1.02** |  |
| **Composite outcomes** | **23 (6.1)** | **3.87 ± 0.97** | **356 (93.9)** | **3.87 ± 1.03** |  |
| **Naming** | | | | |  |
| **Clinical outcome** | **With outcome** | | **Without outcome** | |  |
|  | **n (%)** | **Mean ± SD** | **n (%)** | **Mean ± SD** |  |
| **30 days postoperatively** | | | | |  |
| **Postoperative delirium** | **6 (1.6)** | **2.83 ± 0.41** | **376 (98.4)** | **2.90 ± 0.33** |  |
| **All-cause complications** | **123 (32.2)** | **2.94 ± 0.28** | **259 (67.8)** | **2.88 ± 0.35** |  |
| **Non-home discharge** | **46 (12.1)** | **2.98 ± 0.15** | **335 (87.9)** | **2.89 ± 0.35** |  |
| **ER visits** | **42 (11.0)** | **2.93 ± 0.34** | **339 (89.0)** | **2.90 ± 0.33** |  |
| **30-day readmission** | **10 (2.6)** | **3.00 ± 0.00** | **371 (97.4)** | **2.90 ± 0.33** |  |
| **Mortality** | **2 (0.5)** | **3.00 ± 0.00** | **380 (99.5)** | **2.90 ± 0.33** |  |
| **Composite outcomes** | **146 (38.2)** | **2.95 ± 0.26** | **236 (61.8)** | **2.88 ± 0.37** |  |
| **90 days postoperatively** | | | | |  |
| **All-cause complications** | **20 (5.3)** | **3.00 ± 0.00** | **359 (94.7)** | **2.90 ± 0.34** |  |
| **ER visits** | **14 (3.7)** | **3.00 ± 0.00** | **365 (96.3)** | **2.90 ± 0.33** |  |
| **Mortality** | **2 (0.5)** | **3.00 ± 0.00** | **377 (99.5)** | **2.91 ± 0.33** |  |
| **Composite outcomes** | **23 (6.1)** | **3.00 ± 0.00** | **356 (93.9)** | **2.90 ± 0.34** |  |
| **Attention** | | | | |  |
| **Clinical outcome** | **With outcome** | | **Without outcome** | |  |
|  | **n (%)** | **Mean ± SD** | **n (%)** | **Mean ± SD** |  |
| **30 days postoperatively** | | | | |  |
| **Postoperative delirium** | **6 (1.6)** | **5.17 ± 0.98** | **376 (98.4)** | **5.60 ± 0.79** |  |
| **All-cause complications** | **123 (32.2)** | **5.56 ± 0.83** | **259 (67.8)** | **5.61 ± 0.78** |  |
| **Non-home discharge** | **46 (12.1)** | **5.33 ± 0.97** | **335 (87.9)** | **5.63 ± 0.77** |  |
| **ER visits** | **42 (11.0)** | **5.45 ± 0.89** | **339 (89.0)** | **5.61 ± 0.79** |  |
| **30-day readmission** | **10 (2.6)** | **5.40 ± 1.08** | **371 (97.4)** | **5.60 ± 0.79** |  |
| **Mortality** | **2 (0.5)** | **5.50 ± 0.71** | **380 (99.5)** | **5.60 ± 0.80** |  |
| **Composite outcomes** | **146 (38.2)** | **5.56 ± 0.83** | **236 (61.8)** | **5.61 ± 0.78** |  |
| **90 days postoperatively** | | | | |  |
| **All-cause complications** | **20 (5.3)** | **5.30 ± 0.80** | **359 (94.7)** | **5.61 ± 0.80** |  |
| **ER visits** | **14 (3.7)** | **5.57 ± 0.65** | **365 (96.3)** | **5.60 ± 0.81** |  |
| **Mortality** | **2 (0.5)** | **5.00 ± 1.41** | **377 (99.5)** | **5.60 ± 0.80** |  |
| **Composite outcomes** | **23 (6.1)** | **5.30 ± 0.82** | **356 (93.9)** | **5.62 ± 0.80** |  |
| **Language** | | | | |  |
| **Clinical outcome** | **With outcome** | | **Without outcome** | |  |
|  | **n (%)** | **Mean ± SD** | **n (%)** | **Mean ± SD** |  |
| **30 days postoperatively** | | | | |  |
| **Postoperative delirium** | **6 (1.6)** | **2.67 ± 0.51** | **376 (98.4)** | **2.47 ± 0.77** |  |
| **All-cause complications** | **123 (32.2)** | **2.42 ± 0.82** | **259 (67.8)** | **2.50 ± 0.73** |  |
| **Non-home discharge** | **46 (12.1)** | **2.41 ± 0.78** | **335 (87.9)** | **2.48 ± 0.76** |  |
| **ER visits** | **42 (11.0)** | **2.38 ± 0.70** | **339 (89.0)** | **2.49 ± 0.77** |  |
| **30-day readmission** | **10 (2.6)** | **2.50 ± 0.97** | **371 (97.4)** | **2.47 ± 0.76** |  |
| **Mortality** | **2 (0.5)** | **2.50 ± 0.71** | **380 (99.5)** | **2.48 ± 0.76** |  |
| **Composite outcomes** | **146 (38.2)** | **2.39 ± 0.82** | **236 (61.8)** | **2.53 ± 0.72** |  |
| **90 days postoperatively** | | | | |  |
| **All-cause complications** | **20 (5.3)** | **2.55 ± 0.76** | **359 (94.7)** | **2.47 ± 0.77** |  |
| **ER visits** | **14 (3.7)** | **2.43 ± 1.02** | **365 (96.3)** | **2.48 ± 0.75** |  |
| **Mortality** | **2 (0.5)** | **2.50 ± 0.71** | **377 (99.5)** | **2.48 ± 0.77** |  |
| **Composite outcomes** | **23 (6.1)** | **2.43 ± 0.90** | **356 (93.9)** | **2.48 ± 0.76** |  |
| **Abstraction** | | | | |  |
| **Clinical outcome** | **With outcome** | | **Without outcome** | |  |
|  | **n (%)** | **Mean ± SD** | **n (%)** | **Mean ± SD** |  |
| **30 days postoperatively** | | | | |  |
| **Postoperative delirium** | **6 (1.6)** | **1.83 ± 0.41** | **376 (98.4)** | **1.89 ± 0.36** |  |
| **All-cause complications** | **123 (32.2)** | **1.85 ± 0.40** | **259 (67.8)** | **1.90 ± 0.34** |  |
| **Non-home discharge** | **46 (12.1)** | **1.85 ± 0.36** | **335 (87.9)** | **1.89 ± 0.36** |  |
| **ER visits** | **42 (11.0)** | **1.83 ± 0.38** | **339 (89.0)** | **1.89 ± 0.36** |  |
| **30-day readmission** | **10 (2.6)** | **1.70 ± 0.68** | **371 (97.4)** | **1.89 ± 0.35** |  |
| **Mortality** | **2 (0.5)** | **2.00 ± 0.00** | **380 (99.5)** | **1.88 ± 0.36** |  |
| **Composite outcomes** | **146 (38.2)** | **1.86 ± 0.38** | **236 (61.8)** | **1.90 ± 0.34** |  |
| **90 days postoperatively** | | | | |  |
| **All-cause complications** | **20 (5.3)** | **1.90 ± 0.31** | **359 (94.7)** | **1.88 ± 0.36** |  |
| **ER visits** | **14 (3.7)** | **1.86 ± 0.36** | **365 (96.3)** | **1.89 ± 0.36** |  |
| **Mortality** | **2 (0.5)** | **2.00 ± 0.00** | **377 (99.5)** | **1.88 ± 0.36** |  |
| **Composite outcomes** | **23 (6.1)** | **1.87 ± 0.34** | **356 (93.9)** | **1.89 ± 0.36** |  |
| **Delayed recall** | | | | |  |
| **Clinical outcome** | **With outcome** | | **Without outcome** | |  |
|  | **n (%)** | **Mean ± SD** | **n (%)** | **Mean ± SD** |  |
| **30 days postoperatively** | | | | |  |
| **Postoperative delirium** | **6 (1.6)** | **1.50 ± 1.05** | **376 (98.4)** | **3.16 ± 1.47** |  |
| **All-cause complications** | **123 (32.2)** | **3.20 ± 1.44** | **259 (67.8)** | **3.10 ± 1.50** |  |
| **Non-home discharge** | **46 (12.1)** | **3.02 ± 1.48** | **335 (87.9)** | **3.15 ± 1.48** |  |
| **ER visits** | **42 (11.0)** | **3.43 ± 1.40** | **339 (89.0)** | **3.10 ± 1.49** |  |
| **30-day readmission** | **10 (2.6)** | **3.70 ± 1.34** | **371 (97.4)** | **3.12 ± 1.48** |  |
| **Mortality** | **2 (0.5)** | **3.50 ± 0.71** | **380 (99.5)** | **3.13 ± 1.48** |  |
| **Composite outcomes** | **146 (38.2)** | **3.14 ± 1.41** | **236 (61.8)** | **3.13 ± 1.52** |  |
| **90 days postoperatively** | | | | |  |
| **All-cause complications** | **20 (5.3)** | **3.30 ± 1.17** | **359 (94.7)** | **3.13 ± 1.50** |  |
| **ER visits** | **14 (3.7)** | **3.50 ± 0.94** | **365 (96.3)** | **3.12 ± 1.50** |  |
| **Mortality** | **2 (0.5)** | **4.00 ± 0.00** | **377 (99.5)** | **3.13 ± 1.49** |  |
| **Composite outcomes** | **23 (6.1)** | **3.35 ± 1.11** | **356 (93.9)** | **3.12 ± 1.51** |  |
| **Orientation** | | | | |  |
| **Clinical outcome** | **With outcome** | | **Without outcome** | |  |
|  | **n (%)** | **Mean ± SD** | **n (%)** | **Mean ± SD** |  |
| **30 days postoperatively** | | | | |  |
| **Postoperative delirium** | **6 (1.6)** | **5.50 ± 0.84** | **376 (98.4)** | **5.96 ± 0.20** |  |
| **All-cause complications** | **123 (32.2)** | **5.91 ± 0.34** | **259 (67.8)** | **5.98 ± 0.15** |  |
| **Non-home discharge** | **46 (12.1)** | **5.85 ± 0.42** | **335 (87.9)** | **5.97 ± 0.19** |  |
| **ER visits** | **42 (11.0)** | **5.93 ± 0.26** | **339 (89.0)** | **5.96 ± 0.23** |  |
| **30-day readmission** | **10 (2.6)** | **6.00 ± 0.00** | **371 (97.4)** | **5.95 ± 0.23** |  |
| **Mortality** | **2 (0.5)** | **6.00 ± 0.00** | **380 (99.5)** | **5.96 ± 0.23** |  |
| **Composite outcomes** | **146 (38.2)** | **5.90 ± 0.34** | **236 (61.8)** | **5.99 ± 0.11** |  |
| **90 days postoperatively** | | | | |  |
| **All-cause complications** | **20 (5.3)** | **5.85 ± 0.37** | **359 (94.7)** | **5.96 ± 0.22** |  |
| **ER visits** | **14 (3.7)** | **5.86 ± 0.36** | **365 (96.3)** | **5.96 ± 0.23** |  |
| **Mortality** | **2 (0.5)** | **6.00 ± 0.00** | **377 (99.5)** | **5.96 ± 0.23** |  |
| **Composite outcomes** | **23 (6.1)** | **5.87 ± 0.34** | **356 (93.9)** | **5.96 ± 0.22** |  |

Abbreviations: ER, emergency room; LOS, length of stay. Lower MoCA scores indicates poorer performance. Data on LOS, non-home discharge, ER visit, and readmission were unavailable in one patient at 30 days due to in-hospital mortality. All-cause complications included cardiovascular, respiratory, gastrointestinal, renal, neurological, and surgical complications. Composite adverse outcomes included postoperative delirium, all-cause complications, non-home discharge, ER visits, 30-day readmission and/or mortality.

# **Supplementary Table S12. Associations between poorer MoCA performance on executive/visuospatial function and the incidence of adverse outcomes at 30 and 90 days postoperatively.**

| **Clinical outcomes** | **Univariable Analysis** | | **Multivariable Analysis^a^** | | | | |
| --- | --- | --- | --- | --- | --- | --- | --- |
|  | **Unadjusted estimate**  **(95% CI)** | **Unadjusted P-value** | **Adjusted estimate (95% CI)** | **Adjusted P-value** | **Multiplicity-adjusted P-value**  **(BH within domains)** | | **Multiplicity-adjusted P-value**  **(BH within/across domains)** |
| **30 days** | | | | | | | |
| **Postoperative delirium** | **2.04 (1.00, 4.23)** | **0.047** | **2.41 (1.16, 5.18)** | **0.018** | **0.18** | **0.16** | |
| **All-cause complications^b^** | **1.09 (0.88, 1.34)** | **0.44** | **1.01 (0.81, 1.26)** | **0.91** | **0.96** | **0.96** | |
| **LOS, days^c^** | **0.03 (-0.05, 0.10)** | **0.48** | **-0.002 (-0.078, 0.074)** | **0.96** | **0.96** | **0.97** | |
| **Non-home discharge^c^** | **0.93 (0.68, 1.25)** | **0.63** | **0.87 (0.61, 1.20)** | **0.39** | **0.96** | **0.78** | |
| **ER visits^c^** | **1.04 (0.75, 1.40)** | **0.83** | **0.97 (0.69, 1.35)** | **0.87** | **0.96** | **0.96** | |
| **30-day readmission^c^** | **0.97 (0.50, 1.74)** | **0.92** | **0.90 (0.44, 1.67)** | **0.75** | **0.96** | **0.94** | |
| **Mortality** | **1.37 (0.33, 4.68)** | **0.62** | **-** | **-** | **-** | **-** | |
| **Composite outcomes^d^** | **1.08 (0.88, 1.32)** | **0.47** | **0.99 (0.80, 1.23)** | **0.94** | **0.96** | **0.97** | |
| **90 days** | | | | | | | |
| **All-cause complications^b^** | **0.87 (0.54, 1.35)** | **0.56** | **0.83 (0.50, 1.31)** | **0.44** | **0.96** | **0.78** | |
| **ER visits** | **0.55 (0.27, 1.01)** | **0.08** | **0.48 (0.22, 0.91)** | **0.038** | **0.19** | **0.25** | |
| **Mortality** | **1.38 (0.33, 4.69)** | **0.61** | **-** | **-** | **-** | **-** | |
| **Composite outcomes^d^** | **1.001 (0.651, 1.490)** | **>0.99** | **0.96 (0.61, 1.46)** | **0.86** | **0.96** | **0.96** | |

Abbreviations: ER, emergency room; LOS, length of stay. Estimates expressed as odds ratio for binary outcomes or beta-coefficient for LOS (log-transformed), corresponding to each one unit decrease in MoCA subdomain score (lower scores indicates poorer performance). BH corrections were applied within each MoCA domain (BH within domains) and both within and across MoCA domains (BH within/across domains). ^a^For outcomes of postoperative delirium and 30-day readmission, multivariable analyses were adjusted for education. Mortality was not adjusted due to low count for event. For 30-day outcomes of all-cause complications, length of stay, non-home discharge, emergency room (ER) visits, and composite adverse outcomes, multivariable analyses were adjusted for age, sex, education, and American Society of Anesthesiologists (ASA) physical status. For 90-day outcomes of all-cause complications, ER visits, and composite adverse outcomes, multivariable analyses were adjusted for age and education. ^b^All-cause complications included cardiovascular, respiratory, surgical, gastrointestinal, renal, and neurological complications. ^c^Data on LOS, non-home discharge, ER visit, and readmission were unavailable in one patient at 30 days due to in-hospital mortality. ^d^Composite adverse outcomes included postoperative delirium, all-cause complications, non-home discharge, ER visits, 30-day readmission and/or mortality. P-values of $\leq$0.05 indicate statistical significance.

# **Supplementary Table S13. Associations between poorer MoCA performance on naming and the incidence of adverse outcomes at 30 and 90 days postoperatively.**

| **Clinical outcomes** | **Univariable Analysis** | | **Multivariable Analysis** | | |  |
| --- | --- | --- | --- | --- | --- | --- |
|  | **Unadjusted estimate**  **(95% CI)** | **Unadjusted P-value** | **Adjusted estimate (95% CI)** | **Adjusted P-value** | **Multiplicity-adjusted P-value**  **(BH within domains)** | **Multiplicity-adjusted P-value**  **(BH within/across domains)** |
| **30 days** | | | | | | |
| **Postoperative delirium^a^** | **1.63 (0.10, 7.01)** | **0.61** | **2.10 (0.12, 10.92)** | **0.47** | **0.50** | **0.78** |
| **All-cause complications^b^** | **0.61 (0.27, 1.23)** | **0.20** | **0.55 (0.24, 1.11)** | **0.12** | **0.19** | **0.46** |
| **LOS, days^c^** | **-0.16 (-0.39, 0.07)** | **0.18** | **-0.20 (-0.43, 0.02)** | **0.08** | **0.19** | **0.43** |
| **Non-home discharge^c^** | **0.23 (0.01, 0.99)** | **0.14** | **0.22 (0.01, 0.96)** | **0.13** | **0.19** | **0.46** |
| **ER visits^c^** | **0.74 (0.18, 1.93)** | **0.59** | **0.68 (0.17, 1.80)** | **0.50** | **0.50** | **0.78** |
| **30-day readmission^c^** | **NR** | **NR** | **NR** | **NR** | **NR** | **NR** |
| **Mortality** | **NR** | **NR** | **-** | **-** | **-** | **-** |
| **Composite outcomes^d^** | **0.48 (0.21, 0.97)** | **0.06** | **0.43 (0.18, 0.87)** | **0.029** | **0.17** | **0.21** |
| **90 days** | | | | | | |
| **All-cause complications^b^** | **NR** | **NR** | **NR** | **NR** | **NR** | **NR** |
| **ER visits^c^** | **NR** | **NR** | **NR** | **NR** | **NR** | **NR** |
| **Mortality** | **NR** | **NR** | **-** | **-** | **-** | **-** |
| **Composite outcomes^d^** | **NR** | **NR** | **NR** | **NR** | **NR** | **NR** |

Abbreviations: ER, emergency room; LOS, length of stay; NR, not reported (due to data separation). Estimates expressed as odds ratio for binary outcomes or beta-coefficient for LOS (log-transformed), corresponding to each one unit decrease in MoCA subdomain score (lower scores indicates poorer performance). BH corrections were applied within each MoCA domain (BH within domains) and both within and across MoCA domains (BH within/across domains). ^a^For outcomes of postoperative delirium and 30-day readmission, multivariable analyses were adjusted for education. Mortality was not adjusted due to low count for event. For 30-day outcomes of all-cause complications, length of stay, non-home discharge, emergency room (ER) visits, and composite adverse outcomes, multivariable analyses were adjusted for age, sex, education, and American Society of Anesthesiologists (ASA) physical status. For 90-day outcomes of all-cause complications, ER visits, and composite adverse outcomes, multivariable analyses were adjusted for age and education. ^b^All-cause complications included cardiovascular, respiratory, surgical, gastrointestinal, renal, and neurological complications. ^c^Data on LOS, non-home discharge, ER visit, and readmission were unavailable in one patient at 30 days due to in-hospital mortality. ^d^Composite adverse outcomes included postoperative delirium, all-cause complications, non-home discharge, ER visits, 30-day readmission and/or mortality. P-values of $\leq$0.05 indicate statistical significance.

# **Supplementary Table S14. Associations between poorer MoCA performance on attention and the incidence of adverse outcomes at 30 and 90 days postoperatively.**

| **Clinical outcomes** | **Univariable Analysis** | | **Multivariable Analysis** | | |  |
| --- | --- | --- | --- | --- | --- | --- |
|  | **Unadjusted estimate**  **(95% CI)** | **Unadjusted P-value** | **Adjusted estimate (95% CI)** | **Adjusted P-value** | **Multiplicity-adjusted P-value**  **(BH within domains)** | **Multiplicity-adjusted P-value**  **(BH within/across domains)** |
| **30 days** | | | | | | |
| **Postoperative delirium^a^** | **1.59 (0.67, 3.01)** | **0.20** | **1.75 (0.74, 3.40)** | **0.12** | **0.31** | **0.46** |
| **All-cause complications^b^** | **1.08 (0.82, 1.40)** | **0.57** | **1.05 (0.80, 1.37)** | **0.73** | **0.81** | **0.93** |
| **LOS, days^c^** | **0.04 (-0.05, 0.14)** | **0.37** | **0.04 (-0.06, 0.13)** | **0.45** | **0.71** | **0.78** |
| **Non-home discharge^c^** | **1.47 (1.06, 2.01)** | **0.018** | **1.49 (1.06, 2.06)** | **0.017** | **0.17** | **0.16** |
| **ER visits^c^** | **1.25 (0.85, 1.74)** | **0.22** | **1.24 (0.84, 1.74)** | **0.24** | **0.49** | **0.61** |
| **30-day readmission^c^** | **1.29 (0.59, 2.28)** | **0.44** | **1.26 (0.57, 2.23)** | **0.50** | **0.71** | **0.78** |
| **Mortality** | **1.14 (0.08, 3.67)** | **0.87** | **-** | **-** | **-** | **-** |
| **Composite outcomes^d^** | **1.09 (0.84, 1.40)** | **0.53** | **1.05 (0.81, 1.37)** | **0.71** | **0.81** | **0.92** |
| **90 days** | | | | | | |
| **All-cause complications^b^** | **1.45 (0.89, 2.18)** | **0.10** | **1.46 (0.89, 2.23)** | **0.10** | **0.31** | **0.46** |
| **ER visits^c^** | **1.04 (0.47, 1.82)** | **0.91** | **1.01 (0.45, 1.79)** | **0.98** | **0.98** | **0.98** |
| **Mortality** | **1.77 (0.38, 4.83)** | **0.32** | **-** | **-** | **-** | **-** |
| **Composite outcomes^d^** | **1.45 (0.92, 2.14)** | **0.08** | **1.45 (0.91, 2.16)** | **0.09** | **0.31** | **0.43** |

Abbreviations: ER, emergency room; LOS, length of stay. Estimates expressed as odds ratio for binary outcomes or beta-coefficient for LOS (log-transformed), corresponding to each one unit decrease in MoCA subdomain score (lower scores indicates poorer performance). BH corrections were applied within each MoCA domain (BH within domains) and both within and across MoCA domains (BH within/across domains). ^a^For outcomes of postoperative delirium and 30-day readmission, multivariable analyses were adjusted for education. Mortality was not adjusted due to low count for event. For 30-day outcomes of all-cause complications, length of stay, non-home discharge, emergency room (ER) visits, and composite adverse outcomes, multivariable analyses were adjusted for age, sex, education, and American Society of Anesthesiologists (ASA) physical status. For 90-day outcomes of all-cause complications, ER visits, and composite adverse outcomes, multivariable analyses were adjusted for age and education. ^b^All-cause complications included cardiovascular, respiratory, surgical, gastrointestinal, renal, and neurological complications. ^c^Data on LOS, non-home discharge, ER visit, and readmission were unavailable in one patient at 30 days due to in-hospital mortality. ^d^Composite adverse outcomes included postoperative delirium, all-cause complications, non-home discharge, ER visits, 30-day readmission and/or mortality. P-values of $\leq$0.05 indicate statistical significance.

# **Supplementary Table S15. Associations between poorer MoCA performance on language and the incidence of adverse outcomes at 30 and 90 days postoperatively.**

| **Clinical outcomes** | **Univariable Analysis** | | **Multivariable Analysis** | | |  |
| --- | --- | --- | --- | --- | --- | --- |
|  | **Unadjusted estimate**  **(95% CI)** | **Unadjusted P-value** | **Adjusted estimate (95% CI)** | **Adjusted P-value** | **Multiplicity-adjusted P-value**  **(BH within domains)** | **Multiplicity-adjusted P-value**  **(BH within/across domains)** |
| **30 days** | | | | | | |
| **Postoperative delirium^a^** | **0.66 (0.12, 1.95)** | **0.54** | **0.76 (0.13, 2.27)** | **0.69** | **0.88** | **0.92** |
| **All-cause complications^b^** | **1.14 (0.86, 1.50)** | **0.34** | **1.08 (0.81, 1.43)** | **0.62** | **0.88** | **0.87** |
| **LOS, days^c^** | **-0.02 (-0.13, 0.08)** | **0.64** | **-0.05 (-0.15, 0.05)** | **0.34** | **0.88** | **0.78** |
| **Non-home discharge^c^** | **1.12 (0.75, 1.63)** | **0.56** | **1.15 (0.75, 1.69)** | **0.50** | **0.88** | **0.78** |
| **ER visits^c^** | **1.19 (0.78, 1.74)** | **0.40** | **1.13 (0.74, 1.68)** | **0.55** | **0.88** | **0.82** |
| **30-day readmission^c^** | **0.96 (0.35, 2.03)** | **0.92** | **0.89 (0.33, 1.94)** | **0.80** | **0.88** | **0.95** |
| **Mortality** | **0.96 (0.06, 4.37)** | **0.96** | **-** | **-** | **-** | **-** |
| **Composite outcomes^d^** | **1.27 (1.66, 0.97)** | **0.08** | **1.21 (0.91, 1.59)** | **0.18** | **0.88** | **0.53** |
| **90 days** | | | | | | |
| **All-cause complications^b^** | **0.87 (0.43, 1.55)** | **0.66** | **0.86 (0.42, 1.57)** | **0.66** | **0.88** | **0.91** |
| **ER visits** | **1.09 (0.51, 2.02)** | **0.81** | **1.05 (0.48, 2.01)** | **0.88** | **0.88** | **0.96** |
| **Mortality** | **0.96 (0.06, 4.36)** | **0.97** | **-** | **-** | **-** | **-** |
| **Composite outcomes^d^** | **1.08 (0.60, 1.78)** | **0.78** | **1.07 (0.59, 1.80)** | **0.81** | **0.88** | **0.95** |

Abbreviations: ER, emergency room; LOS, length of stay. Estimates expressed as odds ratio for binary outcomes or beta-coefficient for LOS (log-transformed), corresponding to each one unit decrease in MoCA subdomain score (lower scores indicates poorer performance). BH corrections were applied within each MoCA domain (BH within domains) and both within and across MoCA domains (BH within/across domains). ^a^For outcomes of postoperative delirium and 30-day readmission, multivariable analyses were adjusted for education. Mortality was not adjusted due to low count for event. For 30-day outcomes of all-cause complications, length of stay, non-home discharge, emergency room (ER) visits, and composite adverse outcomes, multivariable analyses were adjusted for age, sex, education, and American Society of Anesthesiologists (ASA) physical status. For 90-day outcomes of all-cause complications, ER visits, and composite adverse outcomes, multivariable analyses were adjusted for age and education. ^b^All-cause complications included cardiovascular, respiratory, surgical, gastrointestinal, renal, and neurological complications. ^c^Data on LOS, non-home discharge, ER visit, and readmission were unavailable in one patient at 30 days due to in-hospital mortality. ^d^Composite adverse outcomes included postoperative delirium, all-cause complications, non-home discharge, ER visits, 30-day readmission and/or mortality. P-values of $\leq$0.05 indicate statistical significance.

# **Supplementary Table S16. Associations between poorer MoCA performance on abstraction and the incidence of adverse outcomes at 30 and 90 days postoperatively.**

| **Clinical outcomes** | **Univariable Analysis** | | **Multivariable Analysis** | | |  |
| --- | --- | --- | --- | --- | --- | --- |
|  | **Unadjusted estimate**  **(95% CI)** | **Unadjusted P-value** | **Adjusted estimate (95% CI)** | **Adjusted P-value** | **Multiplicity-adjusted P-value**  **(BH within domains)** | **Multiplicity-adjusted P-value**  **(BH within/across domains)** |
| **30 days** | | | | | | |
| **Postoperative delirium^a^** | **1.40 (0.08, 6.01)** | **0.73** | **1.73 (0.10, 7.85)** | **0.57** | **0.92** | **0.82** |
| **All-cause complications^b^** | **1.40 (0.78, 2.49)** | **0.25** | **1.31 (0.71, 2.36)** | **0.38** | **0.92** | **0.78** |
| **LOS, days^c^** | **0.04 (-0.18, 0.25)** | **0.73** | **0.02 (-0.20, 0.23)** | **0.88** | **0.92** | **0.96** |
| **Non-home discharge^c^** | **1.33 (0.57, 2.71)** | **0.46** | **1.34 (0.55, 2.86)** | **0.48** | **0.92** | **0.78** |
| **ER visits^c^** | **1.46 (0.62, 2.98)** | **0.33** | **1.43 (0.59, 3.02)** | **0.39** | **0.92** | **0.78** |
| **30-day readmission^c^** | **2.45 (0.63, 6.75)** | **0.12** | **2.29 (0.57, 6.60)** | **0.17** | **0.92** | **0.53** |
| **Mortality** | **NR** | **NR** | **-** | **-** | **-** | **-** |
| **Composite outcomes^d^** | **1.31 (0.74, 2.31)** | **0.35** | **1.19 (0.66, 2.15)** | **0.55** | **0.92** | **0.82** |
| **90 days** | | | | | | |
| **All-cause complications^b^** | **0.87 (0.15, 2.63)** | **0.84** | **0.81 (0.14, 2.53)** | **0.77** | **0.92** | **0.94** |
| **ER visits^c^** | **1.21 (0.21, 3.70)** | **0.78** | **1.09 (0.19, 3.45)** | **0.90** | **0.92** | **0.96** |
| **Mortality** | **NR** | **NR** | **-** | **-** | **-** | **-** |
| **Composite outcomes^d^** | **1.12 (0.28, 2.90)** | **0.84** | **1.06 (0.27, 2.83)** | **0.92** | **0.92** | **0.96** |

Abbreviations: ER, emergency room; LOS, length of stay; NR, not reported (due to data separation). Estimates expressed as odds ratio for binary outcomes or beta-coefficient for LOS (log-transformed), corresponding to each one unit decrease in MoCA subdomain score (lower scores indicates poorer performance). BH corrections were applied within each MoCA domain (BH within domains) and both within and across MoCA domains (BH within/across domains). ^a^For outcomes of postoperative delirium and 30-day readmission, multivariable analyses were adjusted for education. Mortality was not adjusted due to low count for event. For 30-day outcomes of all-cause complications, length of stay, non-home discharge, emergency room (ER) visits, and composite adverse outcomes, multivariable analyses were adjusted for age, sex, education, and American Society of Anesthesiologists (ASA) physical status. For 90-day outcomes of all-cause complications, ER visits, and composite adverse outcomes, multivariable analyses were adjusted for age and education. ^b^All-cause complications included cardiovascular, respiratory, surgical, gastrointestinal, renal, and neurological complications. ^c^Data on LOS, non-home discharge, ER visit, and readmission were unavailable in one patient at 30 days due to in-hospital mortality. ^d^Composite adverse outcomes included postoperative delirium, all-cause complications, non-home discharge, ER visits, 30-day readmission and/or mortality. P-values of $\leq$0.05 indicate statistical significance.

# **Supplementary Table S17. Associations between poorer MoCA performance on delayed recall and the incidence of adverse outcomes at 30 and 90 days postoperatively.**

| **Clinical outcomes** | **Univariable Analysis** | | **Multivariable Analysis** | | |  |
| --- | --- | --- | --- | --- | --- | --- |
|  | **Unadjusted estimate**  **(95% CI)** | **Unadjusted P-value** | **Adjusted estimate (95% CI)** | **Adjusted P-value** | **Multiplicity-adjusted P-value**  **(BH within domains)** | **Multiplicity-adjusted P-value**  **(BH within/across domains)** |
| **30 days** | | | | | | |
| **Postoperative delirium^a^** | **2.02 (1.19, 3.78)** | **0.014** | **2.25 (1.29, 4.42)** | **0.008** | **0.08** | **0.10** |
| **All-cause complications^b^** | **0.96 (0.82,1.10)** | **0.54** | **0.93 (0.79, 1.08)** | **0.33** | **0.52** | **0.78** |
| **LOS, days^c^** | **0.04 (-0.01, 0.09)** | **0.13** | **0.04 (-0.02, 0.09)** | **0.18** | **0.47** | **0.53** |
| **Non-home discharge^c^** | **1.06 (0.86, 1.30)** | **0.58** | **1.09 (0.87, 1.35)** | **0.44** | **0.52** | **0.78** |
| **ER visits^c^** | **0.85 (0.67, 1.07)** | **0.17** | **0.81 (0.63, 1.02)** | **0.08** | **0.40** | **0.43** |
| **30-day readmission^c^** | **0.74 (0.42, 1.16)** | **0.23** | **0.71 (0.40, 1.13)** | **0.19** | **0.47** | **0.53** |
| **Mortality** | **0.83 (0.23, 2.10)** | **0.73** | **-** | **-** | **-** | **-** |
| **Composite outcomes^d^** | **0.99 (0.86, 1.14)** | **0.94** | **0.97 (0.84, 1.12)** | **0.69** | **0.69** | **0.92** |
| **90 days** | | | | | | |
| **All-cause complications^b^** | **0.92 (0.66, 1.25)** | **0.61** | **0.89 (0.63, 1.21)** | **0.46** | **0.52** | **0.78** |
| **ER visits^c^** | **0.83 (0.54, 1.20)** | **0.35** | **0.79 (0.51, 1.15)** | **0.24** | **0.48** | **0.61** |
| **Mortality** | **0.60 (0.11, 1.67)** | **0.43** | **-** | **-** | **-** | **-** |
| **Composite outcomes^d^** | **0.90 (0.66, 1.20)** | **0.48** | **0.87 (0.63, 1.17)** | **0.37** | **0.52** | **0.78** |

Abbreviations: ER, emergency room; LOS, length of stay. Estimates expressed as odds ratio for binary outcomes or beta-coefficient for LOS (log-transformed), corresponding to each one unit decrease in MoCA subdomain score (lower scores indicates poorer performance). BH corrections were applied within each MoCA domain (BH within domains) and both within and across MoCA domains (BH within/across domains). ^a^For outcomes of postoperative delirium and 30-day readmission, multivariable analyses were adjusted for education. Mortality was not adjusted due to low count for event. For 30-day outcomes of all-cause complications, length of stay, non-home discharge, emergency room (ER) visits, and composite adverse outcomes, multivariable analyses were adjusted for age, sex, education, and American Society of Anesthesiologists (ASA) physical status. For 90-day outcomes of all-cause complications, ER visits, and composite adverse outcomes, multivariable analyses were adjusted for age and education. ^b^All-cause complications included cardiovascular, respiratory, surgical, gastrointestinal, renal, and neurological complications. ^c^Data on LOS, non-home discharge, ER visit, and readmission were unavailable in one patient at 30 days due to in-hospital mortality. ^d^Composite adverse outcomes included postoperative delirium, all-cause complications, non-home discharge, ER visits, 30-day readmission and/or mortality. P-values of $\leq$0.05 indicate statistical significance.

# **Supplementary Table S18. Associations between poorer MoCA performance on orientation and the incidence of adverse outcomes at 30 and 90 days postoperatively.**

| **Clinical outcomes** | **Univariable Analysis** | | **Multivariable Analysis** | | |  |
| --- | --- | --- | --- | --- | --- | --- |
|  | **Unadjusted estimate**  **(95% CI)** | **Unadjusted P-value** | **Adjusted estimate (95% CI)** | **Adjusted P-value** | **Multiplicity-adjusted P-value**  **(BH within domains)** | **Multiplicity-adjusted P-value**  **(BH within/across domains)** |
| **30 days** | | | | | | |
| **Postoperative delirium^a^** | **8.94 (2.16, 34.49)** | $\mathbf{<}$**0.001** | **11.38 (2.62, 46.77)** | $\mathbf{<}$**0.001** | **0.004** | **0.031** |
| **All-cause complications^b^** | **3.24 (1.30, 9.30)** | **0.017** | **3.14 (1.25, 8.94)** | **0.020** | **0.035** | **0.16** |
| **LOS, days^c^** | **0.51 (0.18, 0.84)** | **0.003** | **0.50 (0.17, 0.82)** | **0.003** | **0.012** | **0.09** |
| **Non-home discharge^c^** | **4.03 (1.54, 10.66)** | **0.004** | **4.10 (1.46, 11.41)** | **0.006** | **0.014** | **0.10** |
| **ER visits^c^** | **1.57 (0.40, 4.39)** | **0.43** | **1.50 (0.36, 4.40)** | **0.51** | **0.51** | **0.78** |
| **30-day readmission^c^** | **NR** | **NR** | **NR** | **NR** | **NR** | **NR** |
| **Mortality** | **NR** | **NR** | **-** | **-** | **-** | **-** |
| **Composite outcomes^d^** | **6.26 (2.08, 27.27)** | **0.004** | **5.56 (1.89, 23.92)** | **0.006** | **0.014** | **0.10** |
| **90 days** | | | | | | |
| **All-cause complications^b^** | **3.02 (0.78, 8.85)** | **0.06** | **2.57 (0.63, 7.74)** | **0.12** | **0.18** | **0.46** |
| **ER visits^c^** | **2.76 (0.50, 9.03)** | **0.14** | **2.32 (0.40, 7.91)** | **0.23** | **0.26** | **0.61** |
| **Mortality** | **NR** | **NR** | **-** | **-** | **-** | **-** |
| **Composite outcomes^d^** | **2.68 (0.69, 7.74)** | **0.09** | **2.39 (0.59, 7.07)** | **0.15** | **0.19** | **0.50** |

Abbreviations: ER, emergency room; LOS, length of stay; NR, not reported (due to data separation). Estimates expressed as odds ratio for binary outcomes or beta-coefficient for LOS (log-transformed), corresponding to each one unit decrease in MoCA subdomain score (lower scores indicates poorer performance). BH corrections were applied within each MoCA domain (BH within domains) and both within and across MoCA domains (BH within/across domains).  ^a^For outcomes of postoperative delirium and 30-day readmission, multivariable analyses were adjusted for education. Mortality was not adjusted due to low count for event. For 30-day outcomes of all-cause complications, length of stay, non-home discharge, emergency room (ER) visits, and composite adverse outcomes, multivariable analyses were adjusted for age, sex, education, and American Society of Anesthesiologists (ASA) physical status. For 90-day outcomes of all-cause complications, ER visits, and composite adverse outcomes, multivariable analyses were adjusted for age and education. ^b^All-cause complications included cardiovascular, respiratory, surgical, gastrointestinal, renal, and neurological complications. ^c^Data on LOS, non-home discharge, ER visit, and readmission were unavailable in one patient at 30 days due to in-hospital mortality. ^d^Composite adverse outcomes included postoperative delirium, all-cause complications, non-home discharge, ER visits, 30-day readmission and/or mortality. P-values of $\leq$0.05 indicate statistical significance.

# **Supplementary Table S19. Sensitivity analyses examining the association between poorer MoCA performance and adverse outcomes.**

| **Clinical outcomes** | **Original Multivariable Analysis^a^** | | **Sensitivity Analysis A^b^** | | **Sensitivity Analysis B^b^** | | **Sensitivity Analysis C^b^** | | **Sensitivity Analysis D^b^** | |
| --- | --- | --- | --- | --- | --- | --- | --- | --- | --- | --- |
|  | **Adjusted estimate (95% CI)** | **Adjusted P-value** | **Adjusted estimate (95% CI)** | **Adjusted P-value** | **Adjusted estimate (95% CI)** | **Adjusted P-value** | **Adjusted estimate (95% CI)** | **Adjusted P-value** | **Adjusted estimate (95% CI)** | **Adjusted P-value** |
| ***Naming*** |  |  |  |  |  |  |  |  |  |  |
| 30-day composite outcomes | 0.43 (0.18. 0.87) | **0.029** | 0.43 (0.18, 0.87) | **0.028** | 0.44 (0.19, 0.90) | **0.035** | 0.44 (0.19, 0.8) | **0.034** | 0.43 (0.19, 0.87) | **0.030** |
| ***Attention*** |  |  |  |  |  |  |  |  |  |  |
| Non-home discharge | 1.49 (1.06, 2.06) | **0.017** | 1.42 (1.00, 1.96) | **0.042** | 1.46 (1.04, 2.01) | **0.023** | 1.51 (1.07, 2.08) | **0.015** | 1.49 (1.06, 2.06) | **0.017** |
| ***Orientation*** |  |  |  |  |  |  |  |  |  |  |
| 30-day all-cause complications | 3.14 (1.25, 8.94) | **0.020** | 3.17  (1.25, 9.27) | **0.022** | 3.29  (1.30, 9.60) | **0.017** | 3.13  (1.24, 9.11) | **0.022** | 3.23  (1.27, 9.45) | **0.019** |
| LOS, days | 0.50 (0.17, 0.82) | **0.003** | 0.50 (0.17, 0.84) | **0.003** | 0.53 (0.20, 0.86) | **0.002** | 0.50 (0.17, 0.84) | **0.003** | 0.50 (0.17, 0.84) | **0.003** |
| Non-home discharge | 4.10 (1.46, 11.41) | **0.006** | 3.70 (1.38, 10.12) | **0.008** | 4.22  (1.55, 11.69) | **0.004** | 3.59  (1.34, 9.82) | **0.010** | 3.75 (1.40, 10.27) | **0.008** |
| 30-day composite outcomes | 5.56 (1.89, 23.92) | **0.006** | 5.90  (1.93, 26.01) | **0.006** | 6.09 (2.01, 26.76) | **0.005** | 5.78 (1.90, 25.43) | **0.006** | 6.00 (1.97, 26.46) | **0.005** |

Abbreviations: LOS, length of stay; 95% CI, 95% confidence interval. Estimates expressed as odds ratios for binary outcomes or beta-coefficients for the continuous outcome of LOS (log-transformed), corresponding to each one unit decrease in MoCA subdomain score (lower scores indicates poorer performance). All-cause complications included cardiovascular, respiratory, gastrointestinal, renal, neurological, and surgical complications. Composite adverse outcomes included postoperative delirium, all-cause complications, non-home discharge, ER visits, 30-day readmission and/or mortality. ^a^For 30-day outcomes of all-cause complications, length of stay, non-home discharge, and composite adverse outcomes, multivariable analyses were adjusted for age, sex, education, and American Society of Anesthesiologists (ASA) physical status. ^b^In addition to age, sex, and education level, sensitivity analyses adjusted for transient ischemic attack/stroke (Sensitivity Analysis A), hypertension (Sensitivity Analysis B), obstructive sleep apnea (Sensitivity Analysis C), and depression/anxiety (Sensitivity Analysis D) instead of ASA status. P-values of $\leq$0.05 indicated statistical significance.

# **Supplementary Table S20. Sensitivity analyses examining the association between poorer orientation performance and adverse outcomes.**

| **Clinical outcomes** | **Univariable Analysis** | | **Original Multivariable Analysis^a^** | | **Sensitivity Multivariable Analysis^b^** | |
| --- | --- | --- | --- | --- | --- | --- |
|  | **Unadjusted estimate (95% CI)** | **Unadjusted *P*-value** | **Adjusted estimate (95% CI)** | **Adjusted P-value** | **Adjusted estimate (95% CI)** | **Adjusted P-value** |
| Postoperative delirium | 8.94 (2.16, 34.49) | $\boldsymbol{<}$**0.001** | 11.38 (2.62, 46.77) | $\boldsymbol{<}$**0.001** | 8.16 (1.77, 36.38) | **0.004** |
| 30-day all-cause complications | 3.24 (1.30, 9.30) | **0.017** | 3.14 (1.25, 8.94) | **0.020** | 3.51 (1.38, 10.19) | **0.012** |
| LOS, days | 0.51 (0.18, 0.84) | **0.003** | 0.50 (0.17, 0.82) | **0.003** | 0.50 (0.17, 0.82) | **0.003** |
| Non-home discharge | 4.03 (1.54, 10.66) | **0.004** | 4.10 (1.46, 11.41) | **0.006** | 3.78 (1.32, 10.72) | **0.012** |
| 30-day composite outcomes | 6.26 (2.08, 27.27) | **0.004** | 5.56 (1.89, 23.92) | **0.006** | 5.75 (1.93, 24.92) | **0.005** |

Abbreviations: LOS, length of stay; 95% CI, 95% confidence interval. Estimates expressed as odds ratios for binary outcomes or beta-coefficients for the continuous outcome of LOS (log-transformed), corresponding to each one unit decrease in MoCA subdomain score (lower scores indicates poorer performance). All-cause complications included cardiovascular, respiratory, gastrointestinal, renal, neurological, and surgical complications. Composite adverse outcomes included postoperative delirium, all-cause complications, non-home discharge, ER visits, 30-day readmission and/or mortality. ^a^For outcomes of postoperative delirium, multivariable analyses were adjusted for education. For 30-day outcomes of all-cause complications, length of stay, non-home discharge, and composite adverse outcomes, multivariable analyses were adjusted for age, sex, education, and American Society of Anesthesiologists (ASA) physical status. ^b^Preoperative CI status on the MoCA was included as an additional covariate in the sensitivity analyses, P-values of $\leq$0.05 indicated statistical significance.

# **Supplementary Table S21. Sensitivity analyses for unmeasured confounding in the associations between poorer orientation performance and adverse postoperative outcomes.**

| **Outcome** | **Adjusted odds ratio (95% CI)** ^a^ | **E-value**  **(Point estimate)** | **E-value**  **(Lower 95% CI bound)** |
| --- | --- | --- | --- |
| Postoperative delirium | 11.38 (2.62, 46.77) | 22.25 | 4.68 |
| 30-day all-cause complications | 3.14 (1.25, 8.94) | 2.94 | 1.48 |
| Non-home discharge | 4.10 (1.46, 11.41) | 7.67 | 2.28 |
| 30-day composite outcomes | 5.56 (1.89, 23.92) | 4.15 | 2.09 |

Abbreviations: 95% CI, 95% confidence interval. Estimates expressed as odds ratios for binary outcomes, corresponding to each one unit decrease in MoCA orientation score (lower scores indicates poorer performance). All-cause complications included cardiovascular, respiratory, gastrointestinal, renal, neurological, and surgical complications. Composite adverse outcomes included postoperative delirium, all-cause complications, non-home discharge, ER visits, 30-day readmission and/or mortality. ^a^For the outcome of postoperative delirium, multivariable analysis was adjusted for education. For 30-day outcomes of all-cause complications, non-home discharge, and composite adverse outcomes, multivariable analyses were adjusted for age, sex, education, and American Society of Anesthesiologists (ASA) physical status. A larger E-value suggests that an unmeasured confounder would need to be strongly associated with both the exposure and outcome to fully explain the observed association.
